# First-Intention Incisional Wound Healing in Dogs and Cats: A Controlled Trial of Dermapliq and Manuka Honey

**DOI:** 10.3390/vetsci11020064

**Published:** 2024-02-01

**Authors:** Pagona G. Gouletsou, Theodora Zacharopoulou, Vassilis Skampardonis, Stefanos G. Georgiou, Dimitrios Doukas, Apostolos D. Galatos, Eugenia Flouraki, Eleftheria Dermisiadou, Chryssoula Margeti, Mariana Barbagianni, Aikaterini Sideri, Vassiliki Tsioli

**Affiliations:** 1 Clinic of Obstetrics and Reproduction, Faculty of Veterinary Science, School of Health Sciences, University of Thessaly, Trikalon 224, 43100 Karditsa, Greece; 2 Clinic of Surgery, Faculty of Veterinary Science, School of Health Sciences, University of Thessaly, Trikalon 224, 43100 Karditsa, Greece; zacharop@uth.gr (T.Z.); stegeorgiou@vet.uth.gr (S.G.G.); agalatos@vet.uth.gr (A.D.G.); eflouraki@uth.gr (E.F.); eldermis@uth.gr (E.D.); chryssamarg@hotmail.com (C.M.); mabarbag@uth.gr (M.B.); ksideri@vet.uth.gr (A.S.); vtsioli@vet.uth.gr (V.T.); 3 Laboratory of Epidemiology, Biostatistics and Animal Health Economics, Faculty of Veterinary Science, School of Health Sciences, University of Thessaly, Trikalon 224, 43100 Karditsa, Greece; bskamp@uth.gr; 4 Laboratory of Pathology, Faculty of Veterinary Science, School of Health Sciences, University of Thessaly, Trikalon 224, 43100 Karditsa, Greece; ddoukas@uth.gr

**Keywords:** cat, comparison dog–cat, Dermapliq, dog, histology, Manuka honey, RGTA, ultrasonography, wound healing

## Abstract

**Simple Summary:**

In order to improve skin healing, many substances have been used for topical application, honey being one of the oldest. More recently, a medical device, Dermapliq, has been available to treat skin lesions. We assessed these two substances, for use in the sutured skin of dogs and cats, by using cosmetic, clinical, ultrasonographical, and histological evaluation. Comparisons were made between each treatment and control, between the two treatments, and between dogs and cats. The results indicate that Dermapliq improves wound healing; however, Μanuka honey was less beneficial. Healing in cats differs from that of dogs, and cats have better cosmetic and clinical outcomes compared to dogs.

**Abstract:**

This study aimed to compare incisional wound healing in cats and dogs after the topical application of Μanuka honey and a new medical device, Dermapliq. Comparisons were made between each treatment and control, between the two treatments, and between dogs and cats. Twelve cats and twelve dogs were included in this study, and the impact of the two substances was examined through cosmetic, clinical, ultrasonographical, and histological evaluation. The use of Dermapliq in first-intention wound healing achieved a significantly better cosmetic evaluation score and better total clinical score at days 20–41, compared to the control, in both dogs and cats. The ultrasonographically estimated wound area was smaller with Dermapliq compared to the control. Wounds treated with Dermapliq showed histologically less inflammation compared to the control. The use of Manuka honey did not show a significantly better cosmetic score compared to the control. Skin thickening was significantly higher after using Manuka honey compared to the control and so was the total clinical score. However, the median wound area, as was evaluated ultrasonographically, was significantly smaller when wounds were treated with Manuka honey, the difference being more apparent in dogs. Dermapliq was proven to be a better choice in achieving favorable wound healing than Manuka honey in dogs and cats in first-intention healing. In our study, cats had a statistically better cosmetic score and less skin thickening and scar width compared to dogs. Histologically, cats showed significantly less edema, higher inflammation and angiogenesis scores, and lower fibroblast and epidermis thickening scores when compared to dogs.

## 1. Introduction

Primary wound closure is appositional healing in the case of clean skin wounds with a minimal contamination or trauma of the tissues [1,2,3]. The goal of primary wound closure is rapid skin healing, maximizing wound cosmesis with minimal complications, such as wound dehiscence, infection, and pain [4,5]. In animals, the use of sutures for primary wound closure aims to direct skin approximation that promotes quick healing and leads to an acceptable functional and cosmetic result. There are recent studies in dogs and cats regarding the advantages of continuous intradermal suturing even though it is a more difficult and time-consuming technique [6,7,8]. Furthermore, healing differs between dogs and cats, so conclusions derived from studies on dogs cannot be extrapolated to cats. Cats have fewer cutaneous perforating vessels than dogs, leading to slower healing, and they also have less cutaneous perfusion in the first 7 days, leading to 50% less breaking strength in skin sutures [2,9,10,11,12].

Recent research in wound healing has focused on understanding the molecular and cellular mechanisms involved in each phase, as well as developing new therapeutic strategies to improve wound healing outcomes [13]. Wound healing is divided into four overlapping phases: hemostasis, inflammation, proliferation, and remodeling/maturation [13,14].

During hemostasis, endothelial cells release the von Willebrand factor, promoting platelet adhesion and the subsequent release of mediators. These mediators contribute to the formation of a fibrin clot, which seals the injury and halts the bleeding. Concurrently, smooth muscle contraction, triggered by elevated calcium ion levels, causes rapid vessel constriction, reducing blood flow. These processes result in the production of vasoactive compounds that play a role in the vasodilation and relaxation of arterial vessels. This phase typically lasts only a few minutes [13,15].

During the inflammatory phase, mast cells stimulate vasodilation through the release of histamine or serotonin, also releasing mediators such as histamine and TNF-alpha [16]. The neutrophils arrive, recruited from damaged vessels and attracted by interleukin 1, tumor necrosis factor alpha, and bacterial toxins [17]. The activated neutrophils eliminate bacteria and cell debris, creating a favorable environment for wound healing by releasing reactive oxygen species, antimicrobial peptides, and proteolytic enzymes [18]. The leukocytes also release cytokines and growth factors to initiate the proliferative phase. In the late stage of inflammation, a switch of macrophage phenotypes from pro-inflammatory (M1 phagocytic) to anti-inflammatory (M2 pro-regenerative) along with neutrophil apoptosis occurs [19]. Additionally, other cell types such as keratinocytes contribute to this process by releasing inflammatory cytokines [13]. Studies have also shown that Langerhans’ cells, a subpopulation of dendritic cells, rapidly increase in number within the first hour after injury, intervening in the initial phase of wound healing, secreting factors that trigger the proliferation of cells involved in this phenomenon [20]. The duration of this phase averages 0–3 days [15,21]. Other molecules, including cytokines, matrix proteins, and enzymes, are also involved in the inflammatory phase. Among these, chemokines are particularly important as they play a crucial role in attracting neutrophils and lymphocytes to coordinate the early stages of wound healing [22].

During the proliferation phase (duration of 3–12 days), the clot is replaced by connective tissue or granulation tissue, while neovascularization, re-epithelialization, and immunomodulation occur concurrently, lasting for days or weeks [18]. The fibroblasts, in addition to being involved in the formation of granulation tissue, are involved in the regulation of both keratinocyte migration and proliferation other than in angiogenesis. Apart from these cell types, macrophages and mast cells consistently release growth factors that are involved in this process [13,15].

The main processes that distinguish the maturation (or remodeling) phase are collagen restoration and wound contraction, the latter due to myofibroblasts originating from the fibroblasts. The remodeling phase is also mediated by various growth factors that regulate the so-called transitions of the mesenchymal–mesenchymal and endothelial–mesenchymal phenotypes [13]. In addition, these transformations occur through the transforming growth factor beta or the Notch signaling pathways, which inhibit the expression of cadherin in endothelial cells [23,24]. Beta2AR has also been described as a critical molecule that mediates the epithelial–mesenchymal transition process [23,24]. During this stage, the mast cells activate fibroblasts, promoting collagen synthesis; secrete growth factors and cytokines that regulate the transition between fibroblasts and myofibroblasts; and release matrix metalloproteinases, thus initiating the degradation of the extracellular matrix [16]. This leads to a reduction in the formation of the extracellular matrix and modifications to its components. Specifically, type III collagen replaces type I collagen, and elastin, which is absent in the granulation tissue, reappears. The cell death of certain cell types in the granulation tissue constitutes a key event in wound resolution [13]. The duration of this phase ranges from 3 days to 6 months [15,21,25].

Many studies exist about substances that have been applied topically to humans and animals in order to improve skin healing, such as antibiotic ointments, petroleum jelly, bioactive glass, vitamin E, sunscreen, silicone products, cyanoacrylates, corticosteroid ointments, and honey [26,27,28,29,30,31,32,33]. Manuka honey is an inexpensive and effective topically applied agent that promotes wound healing due to its high osmolarity, pH, and nutrient content [29,34,35]. Its main difference from other types of honey, which produce hydrogen peroxide, is that it contains methylglyoxal that offers greater antibacterial activity [34,35,36,37]. Even though the antibacterial effects of honey in wounds are well described, the literature does not convincingly report the effect of honey on healing independent of its antimicrobial effects. The only relative work on the effect of Manuka honey in dogs evaluated the effect of a Manuka honey essential oil hydrogel on acute, full-thickness wounds, healing by second intention [38]. In that study, Manuka honey (UMF16) was combined with tea tree oil, jojoba oil, and pectin of citrus origin into a hydrogel, so its findings cannot be attributed solely to Manuka honey [38].

More recently, Dermapliq has been available for skin lesion treatment [39,40]. Dermapliq is a new medical device based on the concept of the reconstruction of the extracellular matrix by a molecule belonging to the family of the Regenerating Agents (RGTAs). RGTAs (carboxymethylglucose sulfate polymers) are biodegradable polymers engineered to mimic heparan sulfate in the extracellular matrix of damaged tissue. RGTA improves tissue healing in several animal models by stabilizing and protecting heparin-binding growth factors (HBGFs) and matrix proteins [41].

To our knowledge, there are no studies in veterinary literature on the use of Manuka honey and Dermapliq in first-intention wound healing in dogs and cats. Furthermore, only two studies on the differences between dogs and cats on skin healing exist [9,10,11].

The goal of this study was to evaluate the effects of Dermapliq and Manuka honey on parameters of wound healing in a controlled experimental setting, comparing the topical application of Dermapliq, Manuka honey, and standard-of-care dressings (control) on both dogs and cats, in first-intention wound healing. We hypothesized that wound healing would be better and that key processes of wound healing would be enhanced when Dermapliq or Manuka honey is applied to acute, full-thickness, surgically closed wounds in dogs and cats. Furthermore, a comparison of healing variables in first-intention wound healing between dogs and cats was attempted.

## 2. Materials and Methods

### 2.1. Animal Ethics

This study, which was carried out in the research facility area of the Clinic of Surgery, Faculty of Veterinary Science, University of Thessaly, Karditsa, Greece, was approved by the Greek National Animal Ethics Committee (license numbers: 1118/45307 21.3.2017, 411/9.1.2020), which confirmed that it complied with the standards of the national and EU legislation regarding animal experimentation.

### 2.2. Animals

A total of 12 (7 males, 5 females) healthy purpose-bred laboratory Beagle dogs, aged 3–7 years and 12 (9 males, 3 females) healthy purpose-bred laboratory domestic shorthaired cats (DSH), aged 2–6 years, were included in this study. All the animals were housed individually in large cages for the first 2 postoperative months, and afterwards in large pens with outdoor access and plenty of rest areas and hiding places. Both animal species had a standard commercial dry maintenance diet and water was given ad libitum. The experiments were in compliance with the laws of the EU and for the whole study, the pain management and handling were appropriate.

### 2.3. Inclusion Criteria

Physical examination, complete blood count, and serum biochemical analyses in blood and urine appeared to be within normal limits and formed inclusion criteria for each animal’s participation in this study. All cats were FeLV and FIV and all dogs were *Leishmania Infantum*-negative. They were routinely treated for parasites and were vaccinated according to a schedule; no animal in this study received any medication for 60 days prior to the start of the experiment.

### 2.4. Experimental Design

On the day of wound creation and on biopsy days,

(a) Dogs were premedicated with acepromazine (Acepromazine, Alfasan, Woerden, the Netherlands) at 0.03 mg/kg intramuscularly (IM), butorphanol (Dolorex, MSD, Boxmeer, The Netherlands) at 0.1 mg/kg IM, and meloxicam (Metacam, Boehringer Ingelheim, Ingelheim am Rhein, Germany) at 0.1 mg/kg subcutaneously (SC). Anesthesia was induced with intravenous (IV) propofol (propofol, Fresenius Kabi, Athens, Greece) increments until tracheal intubation, and maintained with a mixture of isoflurane (Isoflo, Abbott, Maidenhead, UK) in oxygen.

(b) Cats were premedicated with dexmedetomidine (Dexdomitor, Orion Pharma, Espoo, Finland) at 20 μg/kg IM, and buprenorphine (Bupaq, Neocell, Athens, Greece) at 20μg/kg IM. Anesthesia was induced with IV propofol increments until tracheal intubation, and maintained with a mixture of isoflurane in oxygen.

In both dogs and cats, following the induction of anesthesia, a single dose of cefuroxime (Zinacef, GlaxoSmithKline, Athens, Greece) at 20 mg/kg was administered (IV), while during anesthesia, Lactated Ringer’s solution was administered (IV) (4–5 mL/kg/hour, Lactated Ringer’s, VIOSER, Trikala, Greece).

The animals were positioned in sternal recumbency and the dorsolateral area from the cranial aspect of the thorax to the lumbosacral junction was prepared for aseptic surgery. The same veterinary surgeon performed all operations on dogs and cats. Three 4-cm-long skin and subcutaneous tissue incisions were performed on each side of the trunk, at a distance of 6 cm in dogs and 4 cm in cats from the dorsal midline and 8 cm in dogs and 6 cm in cats apart from each other. On each incision, the subcutaneous tissue and the skin were apposed with continuous subcutaneous and continuous intradermal suture patterns by using 5/0 poliglecaprone 25 (Monocryl, Ethicon Inc., Somerville, NJ, USA).

On both sides, one randomly selected sutured wound was covered with Manuka honey (complete layer of honey, approximately 2–5-mm-thick), (Manuka honey gel, Henry Schein, Melville, NY, USA), one was sprayed with Dermapliq (Dermapliq spray, OTR3, Paris, France), and one was used as the control. Manuka honey and Dermapliq were applied every day, until day 15 postoperatively.

The left-sided wounds were used to evaluate the wound healing through subjective clinical and cosmetic evaluation and ultrasonography evaluation, and the right-sided wounds were used for histological evaluation.

### 2.5. Postoperative Care

In dogs, buprenorphine at 10 μg/kg IM was administered during recovery, every 8 h for 2 days, and also meloxicam at 0.1 mg/kg SC once daily for 5 days, along with consecutive postoperative pain assessment.

In cats, buprenorphine at 10 μg/kg IM was administered during recovery, every 8 h for 2 days, and also meloxicam at 0.1 mg/kg SC on the 1st day and 0.05 mg/kg SC once daily for the next 4 days, along with consecutive postoperative pain assessment.

In addition, in both animal species, an amoxicillin/clavulanic acid combination (Synulox, Haupt Pharma Italy, Latina, Italy) at 20 mg/kg was administered, SC, for 48 h.

After surgery and until the 25th postoperative day, the animals wore Elizabethan collars, and adhesive dressings (Cosmopor, Hartmann Hellas AE, Athens, Greece) were applied to protect the wound site. On the cats, the dressings were further secured with post-surgery cat bodysuits.

The animals were checked three times daily, at which time the bandages were inspected for integrity, placement, strikethrough, soiling, or damage. Dogs additionally underwent environmental enrichment twice daily, unassociated with bandage inspection. All bandage changes and wound treatments were performed using an aseptic technique; wounds and surrounding peri-wound skin were gently cleaned with sterile saline-soaked gauze sponges so as not to disturb the wound. Bandages were changed every day until day 21; wounds were rebandaged as described above.

### 2.6. Cosmetic Evaluation

Two experienced surgeons (P.G.G., V.T.) blindly evaluated the appearance of the wounds from wound photographs taken on days 4, 7, 14, 21, 35, 60, 120 and 150 (Supplementary Files, Figure S1), based on a 1–5 visual analogue scale (1: excellent cosmetic result, 5: bad cosmetic result). The two scores given were added to produce the total cosmetic score of each wound.

### 2.7. Clinical Evaluation

Clinical evaluation was performed every day until the 25th postoperative day, always by the same person. The following parameters were evaluated: skin thickening (in mm, by using a tuberculin skin testing ruler), scar width (in mm, with an electronic caliper), abscessation or inflammation (score 0–3), exudate (score 0–3), and wound dehiscence (in cm), according to the scoring system proposed by Gouletsou et al. [7]. The scoring system is presented in Supplementary Files (Table S1). In case of uneven skin thickening or scar width along the incision, the mean value of five measurements was used.

### 2.8. Ultrasonographic Evaluation

The B-mode real-time ultrasonographic (USG) examination of the skin was performed by means of a real-time ultrasound machine. The ultrasound unit (Longport Digital Scanner [LDS1], Longport International Ltd., Reading, UK) was fitted with a 50.0 MHz polyvinylidene difluoride transducer. Scans perpendicular to the long axis of the incision and the adjacent intact skin were taken, in the middle of the skin incision. The wounds were examined postoperatively every 3 days until day 25, once a week until day 60, on day 120, and finally on day 150. The digitized scans were stored on the associated hard drive and were visualized using a color palette (rainbow). Images were compressed laterally to facilitate viewing of the wound area. The wound area calculations (in mm^2^) were performed by using computer software [8,42,43].

### 2.9. Histological Evaluation

The histological evaluation of the healing process was performed on postoperative days 12, 60, and 150, by collecting samples from the incision of the right side, with a disposable biopsy punch of 8 mm.

The first sample was taken on the 12th postoperative (p.o.) day, 1 cm away from the lower commissure of the incision, whilst the others were taken 1.5 cm apart from the previous one, dorsally.

After the collection of the sample, the wound was closed with a simple interrupted stitch with a poliglecaprone 25 4-0 suture.

The skin samples were immediately bisected, under magnification, perpendicularly to the incision and were fixed in 10% neutral buffered formalin (pH 7.4) for 24 h, dehydrated in a graded series of ethanol and xylene, and embedded in paraffin wax. Two serial 5 mm sections were cut from each paraffin block and stained with hematoxylin and eosin (HE). Each tissue section was examined under a light microscope and a representative digital image of each tissue section was captured, using a NIKON ECLIPSE E200 microscope equipped with the Fi1-L2 Digital System (NIKON, Tokio, Japan). The histological sections were evaluated according to already existing scales [7,32,44,45]. A biopsy sample was taken between the incision lines in order to assess the normal skin at the beginning of this study. Afterwards, the wound area was compared to that of the normal skin at the edge of the biopsy sample.

The following parameters were evaluated: edema (score 0–3), inflammation (score 0–3), presence of fibroblasts (score 0–3), angiogenesis (score 0–3), epidermis thickening (the difference between adjacent healthy epidermis thickness and epidermis thickness at the scar), and scar width score (in μm, and score 0–3). The scoring system is presented in Supplementary Files (Table S2).

### 2.10. Statistical Analysis

All statistical analyses were performed using Stata 17.0 (Stata Statistical Software, College Station, TX, USA), while results were interpreted at the 0.05 level of significance.

Our data were organized in a three-level hierarchical structure design, with repeated measurements over time (level 1) clustered in the method per animal (level 2), clustered in animals (level 3). The between-animal heterogeneity was modeled using an animal-level random intercept, while the observed between-method within-animal heterogeneity was accounted for by including another random intercept for each combination of an animal and method [46].

The potential impact of the rater in the cosmetic evaluation of incision wounds was evaluated initially in a three-level ordinal logistic regression model; the cosmetic evaluation score was the dependent variable, while the rater, postoperative day, incision method, and species were the independent variables. Random effect terms at animal and specific animal–method identifiers were included to account for the aforementioned sources of heterogeneity and dependence. Additionally, the inter-rater agreement in the score of the cosmetic evaluation was tested by calculating a weighted Kappa statistic (Cohen’s κ), adjusting for the degree of variation for ordinal data between two raters.

The final cosmetic score of each incision wound resulted by summing the respective scores of the two raters. In order to investigate the potential effect of the applied treatment method on the incision wounds and any species effect in the above resulting score, a mixed effects ordinal logistic regression model was fit. The sum of the cosmetic evaluation score was the dependent variable, while the p.o. day, incision method, and species were the independent variables. Random effect terms at animal and at specific animal–method identifiers were included.

The clinical evaluation of incision wounds was based on the parameters of skin thickening and the width of the resulting scar over the postoperative period of observation. Both parameters, although continuous in nature, were not normally distributed (Shapiro–Wilk W test *p* < 0.001, W = 0.94 and *p* < 0.001, W = 0.977, respectively). Two individual quantile regression models were used to investigate the effect of applied treatment methods and species in the median of skin thickening and scar width on incision wounds, since no assumptions of parameters’ distribution are imposed. Specifically, the *qreg2* command was used to estimate quantile regression, allowing for adjustment of standard errors and t-statistics that are asymptotically valid under heteroskedasticity and intra-cluster correlation between measurements within the same animal and within the specific animal–treatment combination, using the cluster option [47]. The median value of skin thickening and scar width were the dependent variables in the quantile regression models, while the treatment method, species, and p.o. day were the respective independent ones. In order to maximize the detection of any differences, the above associations were also tested in specific time periods: days 1–7, days 10–17, days 21–35, and day >35 for skin thickening and days 1–19, days 20–34, days 35–42, and days 43–150 for scar width. Further, skin thickening and scar width were each transformed to a scale from 0 to 3, corresponding to the 25th, the 50th, and the 75th, respectively, percentile of the distribution of their total values. The aforementioned scores were summed to create a total score for clinical evaluation (total clinical score), ranging from 0 to 6 with a median value of 2 (Shapiro–Wilk W test for normal data *p* = 0.00097, W = 0.99606). Similarly, a non-parametric method for estimating differences in the total clinical score between applied treatments was implemented through quantile regression, in a model structure as described earlier. The median value of the total clinical score was the dependent variable in the quantile regression model, while the treatment, species, and p.o. day were the respective independent ones.

Quantile regression, as previously specified, was also used to investigate the effect of applied treatments in the median of USG evaluation measurement, since no assumptions of parameter distribution are imposed. The median value of USG evaluation was the dependent variable in the quantile regression model, while the treatment, species, and p.o. day were the respective independent ones.

Further, the USG evaluation outcome was also transformed to a scale from 0 to 3, corresponding to the 25th, the 50th, and the 75th, respectively, percentile of the distribution of total values. The total cosmetic score, total clinical score, and USG score were added to create a total score for incision treatment evaluation, ranging from 2 to 14 with a median value of 5.

Quantile regression was used to investigate the effect of applied treatments in the median total score, using the *qreg2* command with the cluster option. The median value of the total score was the dependent variable in the quantile regression model, while the treatment, species, and p.o. day were the respective independent ones.

For the histological evaluation of incision wounds, data regarding the presence of edema, inflammation, fibroblasts, angiogenesis, and epidermis thickening were recorded. The former three parameters were recorded as discrete scores (0 to 3), while the fourth was recorded as a binary variable. Thus, the appropriate way to investigate any differences in the odds of edema, inflammation, and fibroblast scoring between the applied methods and species was in respective mixed effect ordinal logistic regression models, adjusted in a three-level hierarchical design. Edema, inflammation, and fibroblast scores were the dependent variables while the applied method of treatment after incision, species, and a dummy variable encoding the three time points of histological evaluation postoperatively were the independent ones. The effect of the applied treatment methods in the occurrence of angiogenesis was investigated in a mixed effect logistic regression model, adjusted in a three-level hierarchical data structure. Angiogenesis occurrence was the dependent variable while the applied method of treatment after incision and a dummy variable encoding time points of inflammation evaluation were the independent ones. Random effect terms for the animal and for a specific animal–treatment combination level were incorporated in all the above models.

Epidermis thickening was not normally distributed (Shapiro–Wilk test for normality W = 0.806, *p* < 0.0001); thusly, a non-parametric method for estimating differences in this parameter between applied treatments should be implemented.

Quantile regression was used to investigate the effect of applied treatments in the median value of epidermis thickening, using the *qreg2* command with the cluster option. The median value of epidermis thickening was the dependent variable in the quantile regression model, while the treatment, species, and p.o. day were the respective independent ones.

The epidermis thickness evaluation outcome was also transformed to a score on a scale from 0 to 3, corresponding to the 25th, the 50th, and the 75th, respectively, percentile of the distribution of total values.

The scar score was a four-level discrete variable. Thusly, the appropriate way to investigate any differences in the odds of scar score evaluation categories between the applied methods was a mixed effect ordinal logistic regression, adjusted in a three-level design. The scar evaluation score (0 to 3) was the dependent variable while the applied method of treatment after incision, species, and a dummy variable encoding time points of inflammation evaluation, after the application of the incision, were the independent ones. Scar scoring was performed on p.o. days 12, 60, and 150 after incision. Random effect terms for the animal and for a specific animal–treatment combination level were incorporated.

Scores of each of the parameters of histological evaluation (namely edema, inflammation, fibroblasts, angiogenesis, epidermis thickening, and scar score) were added to create a total histological score, ranging from 2 to 14 with a median value of 6. Since the total histological score was not normally distributed (Shapiro–Wilk test for normality W = 0.9589, *p* = 0.00004), the aforementioned non-parametric method of quantile regression for the investigation of the effect of applied treatments in the median of the total histological score was employed, using the *qreg2* command along with the cluster option. The median value of the total histological score was the dependent variable in the quantile regression model, while the treatment, species, and p.o. day were the respective independent ones.

## 3. Results

### 3.1. Cosmetic Evaluation

**Figure 1:**
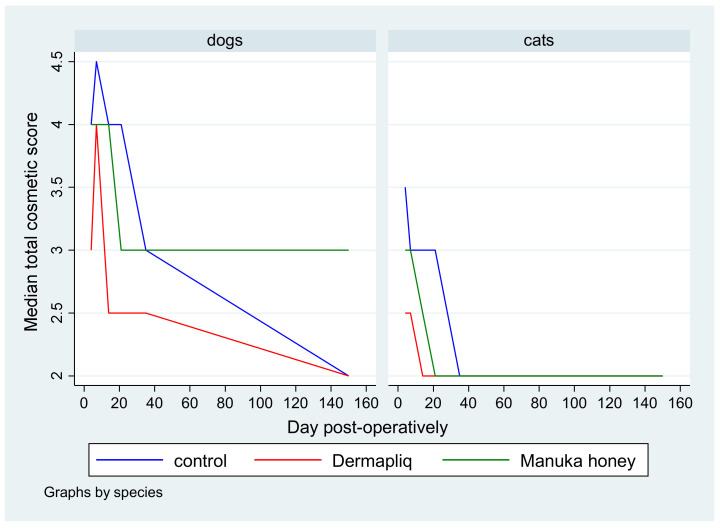
Median total cosmetic score by treatment and species over postoperative days.

**Figure 2:**
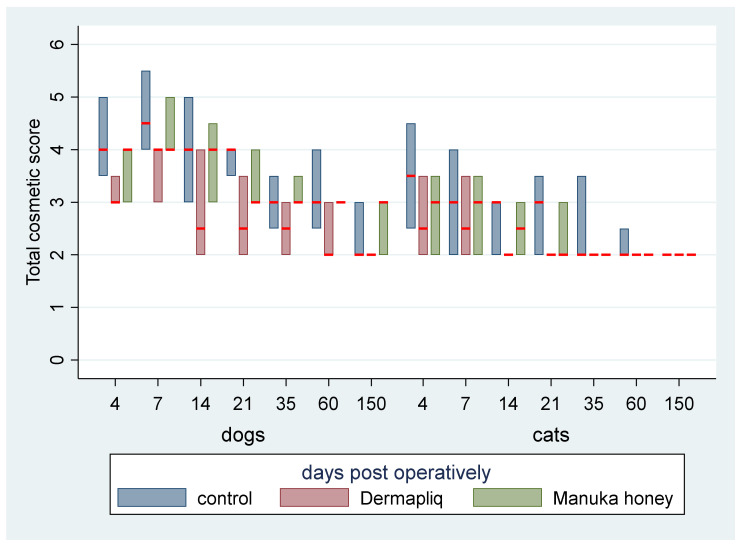
Evaluation of total cosmetic score (median: red line; IQR: column) by treatment and species over postoperative days.

**Figure 3:**
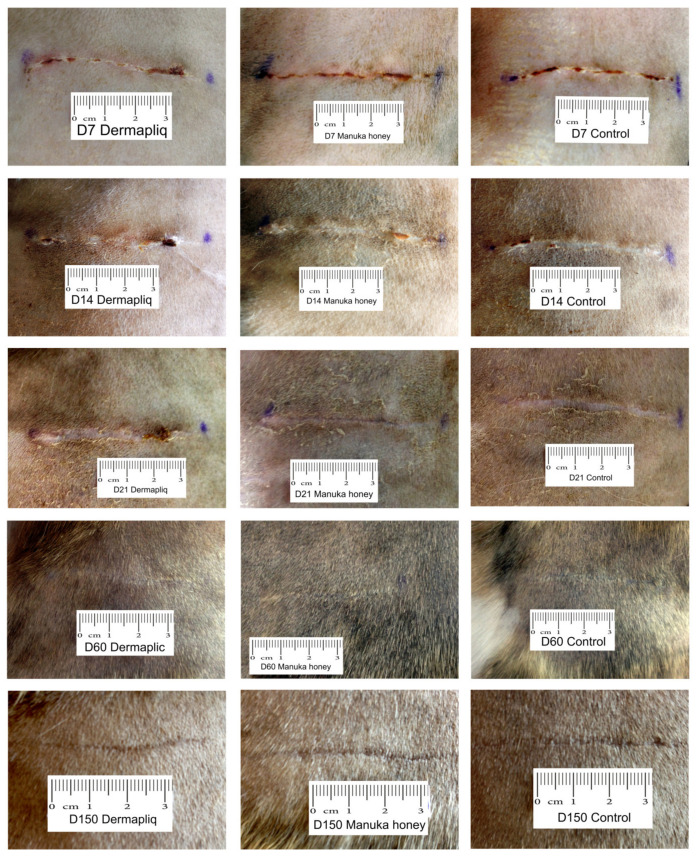
Photographs of the wounds from cat 6.

**Figure 4:**
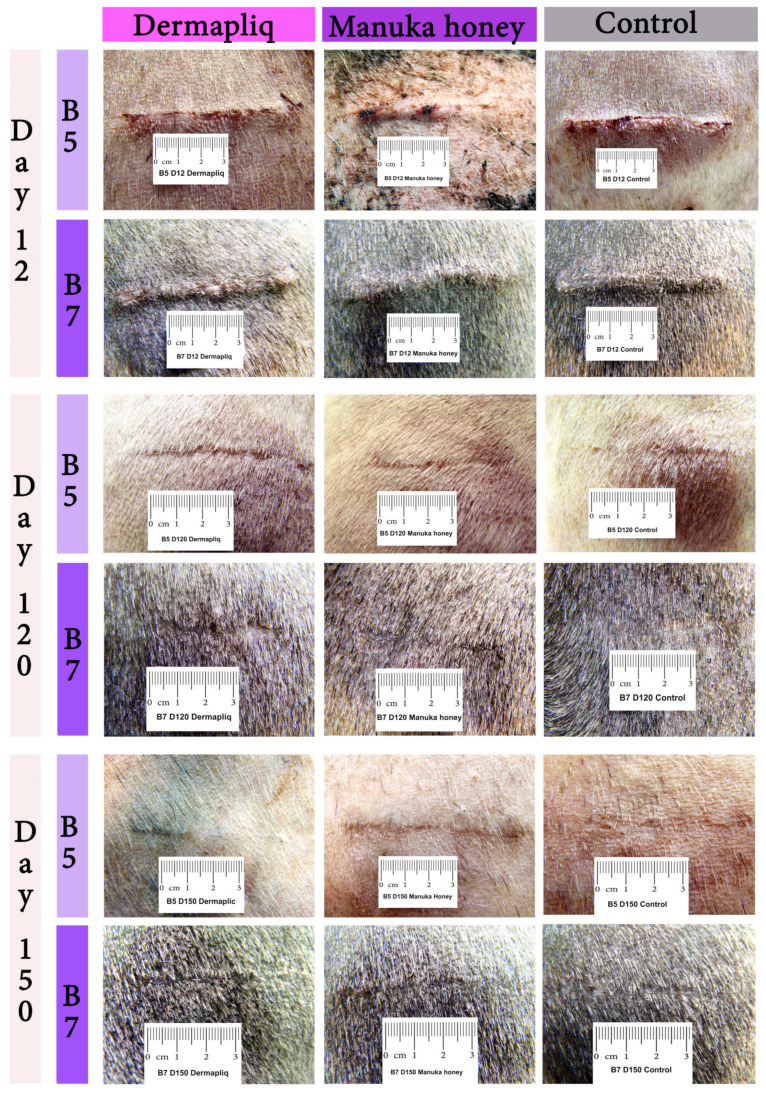
Photographs of the wounds from Beagles 5 and 7.

No difference was found in the odds between cosmetic score categories between the two raters (*p* = 0.751), accounting for the time, treatment, and species effect. Afterwards, the cosmetic scores of the two raters were summed and a new “Total cosmetic score” variable was created. The higher the score, the worse the appearance of the wound in cosmetic evaluation. The median values of total cosmetic scores are presented in Figure 1, whilst the interquartile range (IQR) is presented in Figure 2. Photographs of the wounds are presented in Figure 3 (cats) and Figure 4 (dogs).

A statistically significant difference in the odds of total cosmetic score categories between Dermapliq and the control was observed (*p* < 0.001). Specifically, incisions treated with Dermapliq were 7.28 (95% CI: 3.782; 14) times less likely to have a higher versus lower total cosmetic score, compared to those left untreated. Additionally, a statistically significant difference between Dermapliq and Manuka honey was also observed (*p* = 0.003). Incisions treated with Dermapliq were 4 (95% CI: 2.11; 7.6) times less likely to have a higher versus lower total cosmetic score, compared to those treated with Manuka honey. However, no statistically significant difference between Manuka honey and the control (*p* = 0.052) was observed.

A statistically significant difference in the odds of the cosmetic sum score was observed between species (*p* < 0.001). Cats were 11.6 (95% CI: 6.67; 20) times less likely to have a higher versus lower total cosmetic score compared to dogs.

### 3.2. Clinical Evaluation

All animals tolerated surgery, bandages, and dressing changes well and without complications. All incisions healed without any sign of inflammation, exudate, or abscessation and no wound dehiscence was noticed in any animal.

#### 3.2.1. Skin Thickening

**Figure 5:**
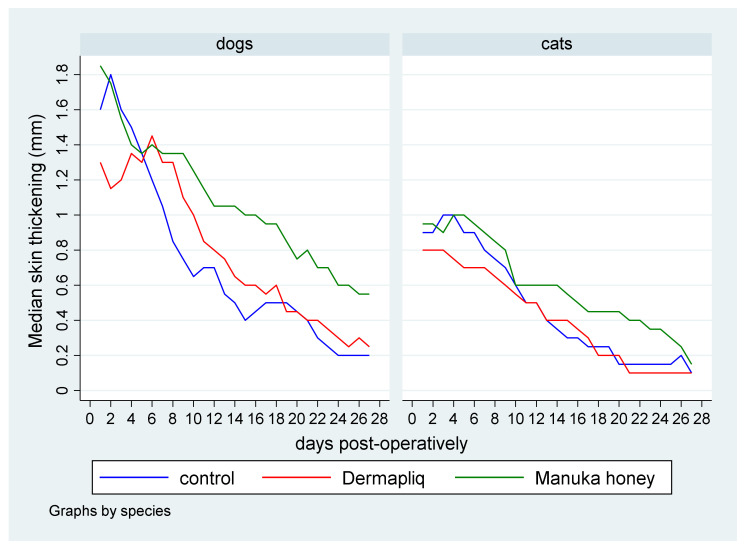
Median skin thickening (in mm) by treatment and species over postoperative days.

The median skin thickening (in mm) by treatment and species over postoperative days is presented in Figure 5.

Statistically significant differences in the median value of skin thickening were detected between Manuka honey and the control (*p* = 0.004) and between Manuka honey and Dermapliq (*p* < 0.001), but not between Dermapliq and the control (*p* = 0.551). Specifically, incision wounds treated postoperatively with Manuka honey had a difference (higher) of 0.30 (95% CI: 0.094; 0.50) and 0.24 (95% CI: 0.11; 0.38) in their median value of skin thickening compared to the control and Dermapliq, respectively. Cats had a difference (lower) of −0.37 (95% CI: −0.6; −0.14) in their median value of skin thickening compared to dogs (*p* = 0.002).

The above associations for skin thickening were tested in specific time periods: days 0–10, 11–19, 20–34, 35–42, and 43–150.

**Figure 6:**
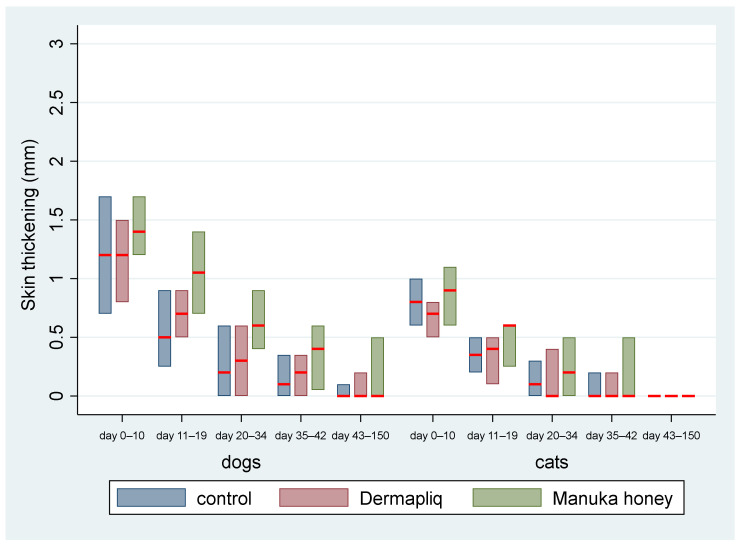
Median skin thickening (red line) and interquartile range (column) by treatment and species over postoperative time periodss.

The median and interquartile range (IQR) of skin thickening by treatment and species over specific postoperative time periods are presented in Figure 6.

- Days 0–10: There was no difference in the median value of skin thickening between the control and Dermapliq (*p* = 0.335), and between the control and Manuka honey (*p* = 0.213), but there was a statistically significant one between Manuka honey and Dermapliq (*p* = 0.001). Specifically, there was a difference (higher) of 0.23 (95% CI: 0.096; 0.37) in the median value of skin thickening of wounds treated with Manuka honey compared to those treated with Dermapliq. Cats had a difference (lower) of −0.51 (*p* < 0.001, 95% CI: −0.7; −0.31) in the median skin thickening compared to dogs, controlling for the treatment and time effect.
- Days 11–19: A statistically significant difference was detected between Manuka honey and the control (*p* = 0.001); specifically, there was a difference (higher) of 0.27 (95% CI: 0.103; 0.43) in the median value of skin thickening of wounds treated with Manuka honey compared to the control. Similarly, a statistically significant difference was detected between Dermapliq and Manuka honey (*p* = 0.001); specifically, there was a difference (higher) of 0.23 (95% CI: 0.09; 0.37) in the median value of skin thickening of wounds treated with Manuka honey compared to those treated with Dermapliq. There was no difference in the median value of skin thickening between the control and Dermapliq (*p* = 0.682). Cats had a difference, controlling for treatment and time, of −0.3 (lower) (*p* = 0.001, 95% CI: −0.47; −0.12) in the skin thickening compared to dogs.
- Days 20–34: The differences in the median value of skin thickening between Manuka honey and the control (*p* = 0.003) and between Dermapliq and Manuka honey (*p* = 0.025) were statistically significant, unlike the one between the control and Dermapliq (*p* = 1.00). Specifically, there was a difference (higher) of 0.3 (95% CI: 0.1; 0.5) and 0.3 (95% CI: 0.038; 0.56) in the median value of skin thickening of wounds treated with Manuka honey compared to the control and Dermapliq, respectively.
- Days 35–42: There was no difference in the median value of skin thickening between methods (all *p* > 0.452).
- Days 42–150: There were no differences at all. All median values were 0.

#### 3.2.2. Scar Width

**Figure 7:**
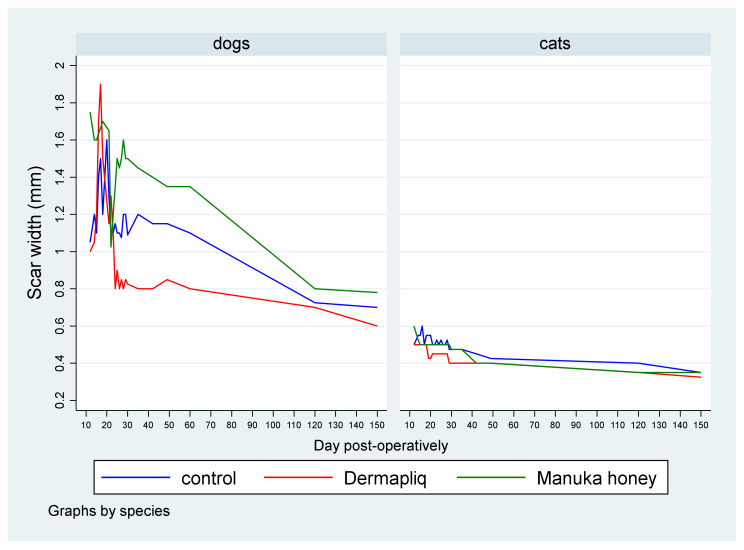
Median scar width (in mm) by treatment and species over postoperative days.

The median scar width (in mm) by treatment and species over postoperative days is presented in Figure 7.

A statistically significant difference between Dermapliq and Manuka honey (*p* = 0.036), adjusting for the time and species effect, was detected. Specifically, incision wounds treated with Manuka honey had a difference (higher) of 0.074 in their median scar width values (95% CI: 0.005; 0.144) compared to those treated with Dermapliq. Cats had a statistically significant difference (lower) in the median value of scar width compared to dogs (*p* < 0.001); specifically, they had a difference of −0.65 (95% CI: −0.86; −0.44).

**Figure 8:**
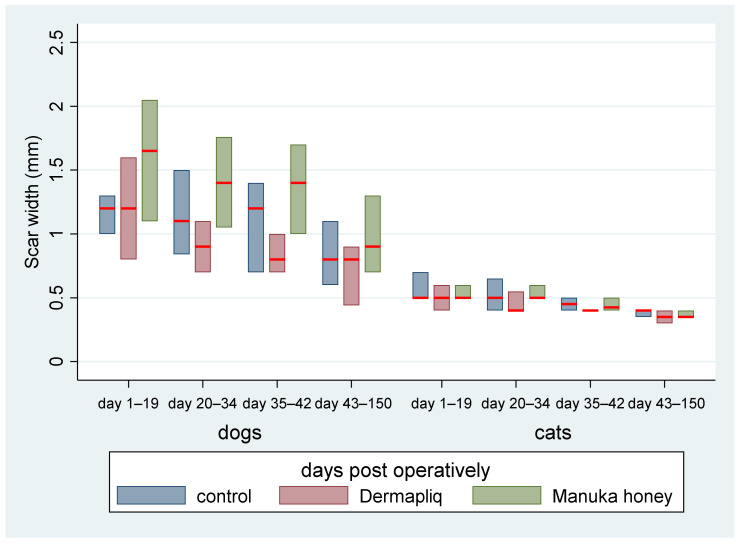
Median (red line) and interquartile range (column) of scar width by treatment and species over specific postoperative time periods.

The above associations for scar width were tested in specific time periods: days 1–19, 20–34, 35–42, and 43–150. The median and interquartile range (IQR) of scar width by treatment and species over specific postoperative time periods are presented in Figure 8.

- Days 1–19: There was no difference in the median value of scar width between methods (all *p* > 0.06). There was a statistically significant effect of species in the median value of scar width (*p* < 0.001). Specifically, cats had a lower difference of −0.8 (95% CI: −0.996; −0.604) in their median scar width value compared to dogs.
- Days 20–34: The differences between Manuka honey and Dermapliq (*p* < 0.001) and between Manuka honey and the control (*p* < 0.001) were significant; incision wounds treated with Manuka honey had a difference (higher) of 0.6 (95% CI: 0.27; 0.93) and 0.4 (95% CI: 0.217; 0.583) in the median value of scar width compared to those treated with Dermapliq and the control, respectively. However, there was no difference in the median value of scar width between Dermapliq treatment and the control (*p* = 0.253). Cats had a difference (lower) of −0.6 (95% CI: −0.78; −0.42) in the median scar width compared to dogs (*p* < 0.001). A statistically significant interaction was detected between species and treatment (*p* = 0.004). The effect of treatment on the median value of scar width was different in cats compared to in dogs. Specifically, the Manuka honey effect, compared to the control, was higher in cats than in dogs by an additional difference of −0.4 (95% CI: −0.6; −0.2) in the median value of scar width, while the Manuka honey effect, compared to Dermapliq, was also higher in cats than in dogs by an additional difference of −0.5 (95% CI: −0.84; −0.16).
- Days 35–42: There was no difference in the median value of scar width between methods (all *p* > 0.093). Cats had a difference (lower) of −0.65 (95% CI: −0.997; −0.303) in the median scar width compared to dogs (*p* < 0.001).
- Days 43–150: There was no difference in the median value of scar width between methods (all *p* > 0.474). Cats had a statistically significant difference (lower) in the median value of scar width compared to dogs (*p* < 0.001); specifically, they had a difference of −0.47 (95% CI: −0.64; −0.3).

#### 3.2.3. Total Clinical Score

**Figure 9:**
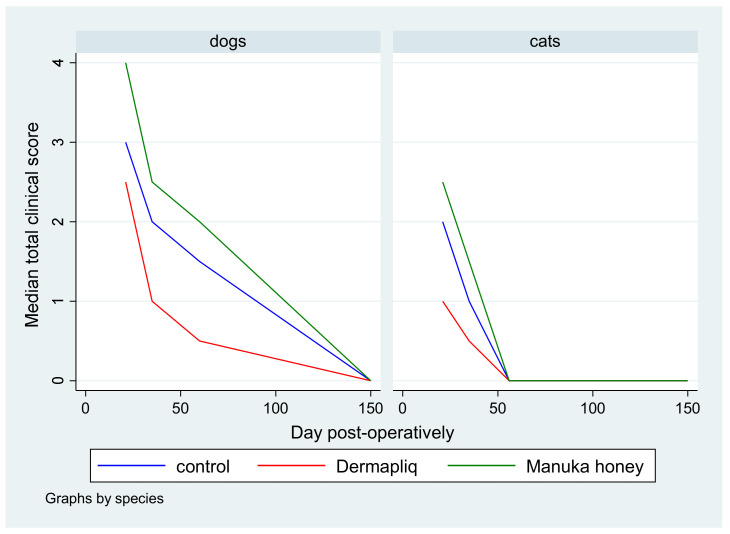
Median total clinical score by treatment and species over postoperative days.

Skin thickening and scar width were each transformed to a scale from 0 to 3. These scores were summed to create a total score for clinical evaluation (total clinical score), ranging from 0 to 6 with a median value of 2. The median total clinical score by treatment and species over postoperative days is presented in Figure 9.

According to the median regression model, there was not any statistically significant difference in the median value of the total clinical score between Dermapliq treatment and the control (*p* = 0.651), whereas the respective differences between Manuka honey treatment and the control (*p* = 0.011) and between Manuka honey and Dermapliq treatment (*p* < 0.001) were statistically significant. Specifically, incision wounds treated with Manuka honey had a difference of 0.94 (95% CI: 0.22; 1.65) and 1.09 (95% CI: 0.48; 1.71) in the median total clinical score compared to controls and those treated with Dermapliq, respectively, controlling for the time and species effect. There was not any significant difference between species (*p* = 0.351). A significant effect of time (postoperative days) was detected (*p* < 0.001) (Coef.: −0.015, 95% CI: −0.021; −0.009), suggesting a 0.015 unit decrease in the median value of the total clinical score for each elapsed day.

**Figure 10:**
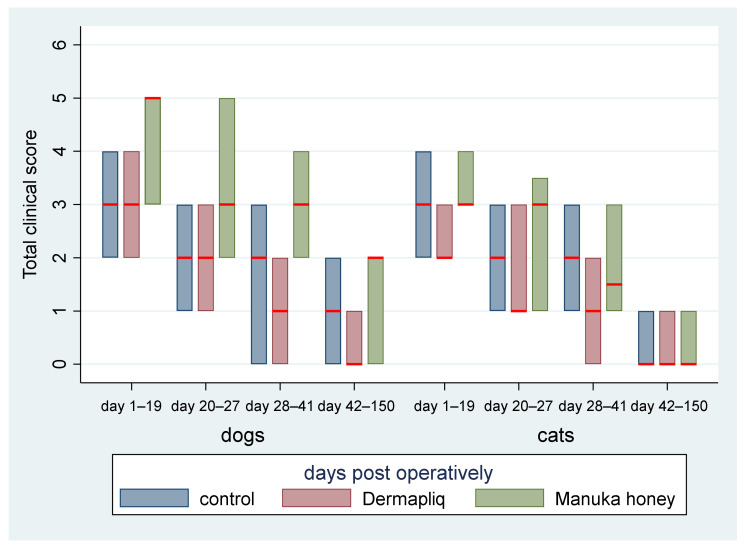
Median (red line) and interquartile range (column) of total clinical score by treatment and species over specific postoperative time periods.

The median and interquartile range (IQR) of the total clinical score by treatment and species over specific postoperative time periods are presented in Figure 10.

The above associations for the total clinical score were tested in specific time periods as well: from day 1 to 19, from day 20 to 27, from day 28 to 41, and from day 42 to 150.

- Days 1–19: There was a statistically significant difference in the median total clinical score between Manuka honey and the control (*p* < 0.001) and Manuka honey and Dermapliq (*p* < 0.001), but not between Dermapliq and the control (*p* = 0.854). Specifically, incision wounds treated with Manuka honey had a difference (higher) of 2 (95% CI: 1.21; 2.78) and 1.9 (95% CI: 1.06; 2.7) in the median total clinical score compared to those treated with the control and Dermapliq, respectively, controlling for the time and species effect. The species effect was significant (*p* < 0.001); specifically, cats had a difference (lower) of −1.7 (95% CI: −2.53; −0.87) in their median value of the total clinical score compared to dogs.
- Days 20–27: There was a statistically significant difference in the median total clinical score, adjusting for the time and species effect, between Dermapliq and the control (*p* = 0.049) and between Dermapliq and Manuka honey (*p* = 0.002), but not between Manuka honey and the control (*p* = 0.176). Specifically, incision wounds treated with Dermapliq had a difference of −0.84 (95% CI: −1.69; −0.002) in the median total clinical score compared to the control. Similarly, incision wounds treated with Manuka honey had a difference of 1.46 (95% CI: 0.52; 2.4) in the median total clinical score compared to Dermapliq. No statistically significant difference between species was detected (*p* = 0.252).
- Days 28–41: There was no difference in the median value of the total clinical score between Manuka honey and the control (*p* = 0.596) and between Dermapliq and Manuka honey (*p* = 0.089). However, the difference between Dermapliq and the control (*p* = 0.031) was significant. Specifically, control incisions had a difference of 1 (95% CI: 0.094; 1.9) in the median total clinical score compared to Dermapliq, controlling for the time and species effect. No statistically significant difference between species was detected (*p* = 0.110).
- Days 42–150: Significant differences between Manuka honey and the control (*p* < 0.001) and between Dermapliq and Manuka honey (*p* < 0.001) were detected. Specifically, incision wounds treated with Manuka honey had a difference of 1.06 (95% CI: 0.73; 1.38) and 1.39 (95% CI: 0.85; 1.92) in the median value of the total clinical score compared to Dermapliq and the control, respectively, controlling for the time and species effect. Nevertheless, there was no difference in the median value of the total clinical score between Dermapliq and the control (*p* = 0.089). A statistically significant difference between species was also detected (*p* < 0.001); specifically, cats had a difference of −1.58 (95% CI: −2.18; −0.98) in their median value of the total clinical score compared to dogs.

### 3.3. Ultrasonographic Evaluation

**Figure 11:**
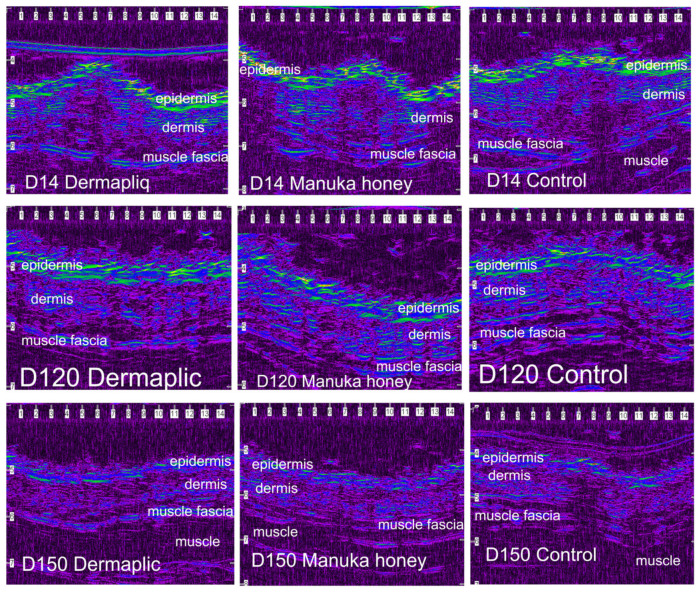
Ultrasound scans of the wound area in a cat. The image is compressed laterally and is visualized using a color palette (rainbow) to facilitate viewing of the wound area (scale in mm).

**Figure 12:**
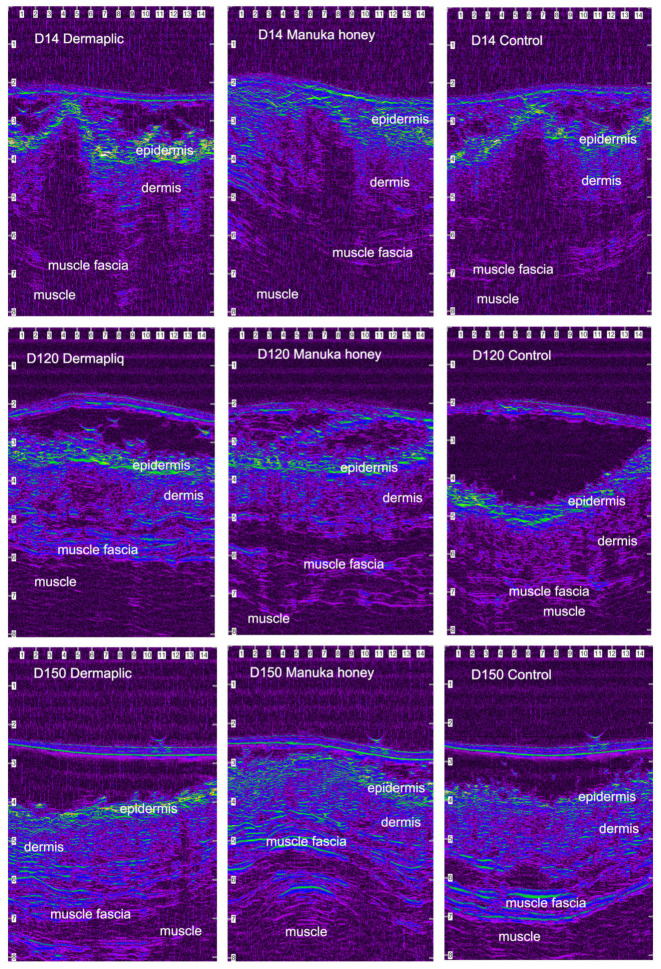
Ultrasound scans of the wound area in a dog. The image is compressed laterally and is visualized using a color palette (rainbow) to facilitate viewing of the wound area (scale in mm).

After wound closure, the ultrasonographic (USG) image of the skin at the wound area differed from the adjacent normal one. The shape of the epidermis had been deformed, creating a cone that protruded 1–2 mm. The area of the dermis at the incision site was enlarged and hypoechogenic compared to the normal adjacent one. The ultrasound scans of the wound area are shown in Figure 11 (cats) and Figure 12 (dogs).

According to the median regression models, there was a statistically significant difference in the median value of USG evaluation between Dermapliq and the control (*p* = 0.019, Coef.: −0.69, 95% CI: −1.26; −0.16) and between Manuka honey and the control (*p* < 0.001, Coef.: −0.99, 95% CI: −0.45; −1.54), but not between Dermapliq and Manuka honey (*p* = 0.189), adjusting for the time and species effect. There was no statistically significant difference in the median value of USG evaluation between species (*p* = 0.519).

**Figure 13:**
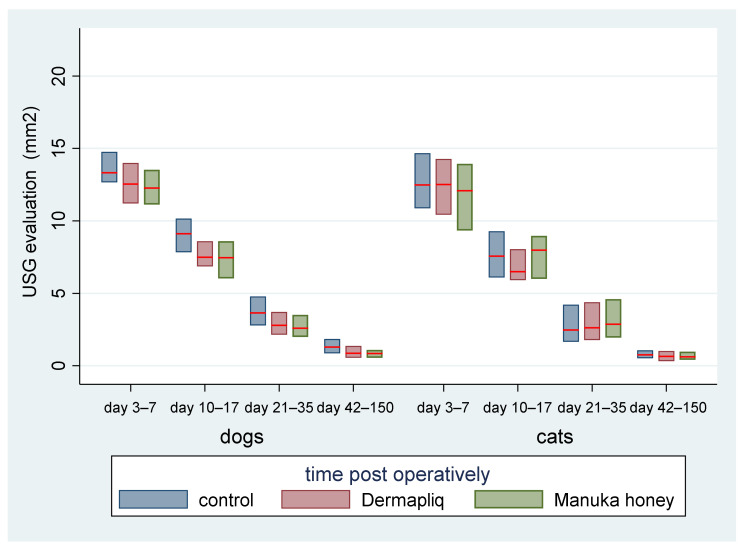
Median (red line) and interquartile range (column) of USG evaluation (in mm^2^) by treatment and species over postoperative time periods.

The above associations for USG evaluation were also tested in specific time periods: days 3 7, 10–17, 21–35, and 42–150. The median and interquartile range (IQR) of USG evaluation (in mm^2^) by treatment and species over postoperative days are presented in Figure 13.

- Days 3–7: There was no statistically significant difference in the median value of USG evaluation between the treatments (all *p* > 0.076).
- Days 10–17: There was a statistically significant difference in the median value of USG evaluation between Dermapliq and the control (*p* = 0.011), but not between the control and Manuka honey (*p* = 0.078) and between Dermapliq and Manuka honey (*p* = 0.647). Specifically, non-treated control wounds had a difference of 1.28 (95% CI: 0.3; 2.26) in the median value of USG evaluation compared to those treated with Dermapliq, controlling for the time and species effect. A statistically significant difference between species was also observed (*p* = 0.024); cats had a difference (lower) of −1.03 (95% CI: −1.92; −014).
- Days 21–35: There was a significant difference in the median value of USG evaluation between Manuka honey and the control (*p* < 0.001) and between Dermapliq (*p* < 0.001) and the control. Specifically, incision wounds treated with Manuka honey had a difference (lower) of −0.87 (95% CI: −1.33; −0.42) in the median value of USG evaluation compared to the control, while Dermapliq-treated incisions had a difference (lower) of −0.71 (95% CI: −1.04; −0.37) compared to controls. The difference in the median value of USG evaluation between Manuka honey and Dermapliq was not statistically significant (*p* = 0.341). Cats had a difference (lower) of −1.43 (95% CI: −2.31; −0.55) in the median USG value compared to dogs.
- Days 42–150: There was a significant difference in the median value of USG evaluation between Dermapliq and the control (*p* < 0.001), and Manuka honey and the control (*p* < 0.001), but not between Dermapliq and Manuka honey (*p* = 0.576). Specifically, Dermapliq had a difference (lower) of −0.41 (95%CI: −0.56; −0.26) in the median value of USG evaluation compared to the control. Manuka honey had a difference (lower) of 0.44 (95%CI: −0.62; −0.26) in the median value of USG evaluation compared to the control. Cats had a difference (lower) of −0.16 (95% CI: −0.31; −0.015) in the median USG value compared to dogs. No significant interactions between time and treatment (*p* = 0.220), and time and species (*p* = 0.07), were observed, but the one between species and treatment was significant (*p* = 0.005), suggesting that a control wound in cats had a lower median value by 0.35 units (95% CI: −0.56; −0.14).

Further, the USG evaluation outcome was also transformed to a scale from 0 to 3, corresponding to the 25th, the 50th, and the 75th, respectively, percentile of the distribution of total values.

### 3.4. Total Score

**Figure 14:**
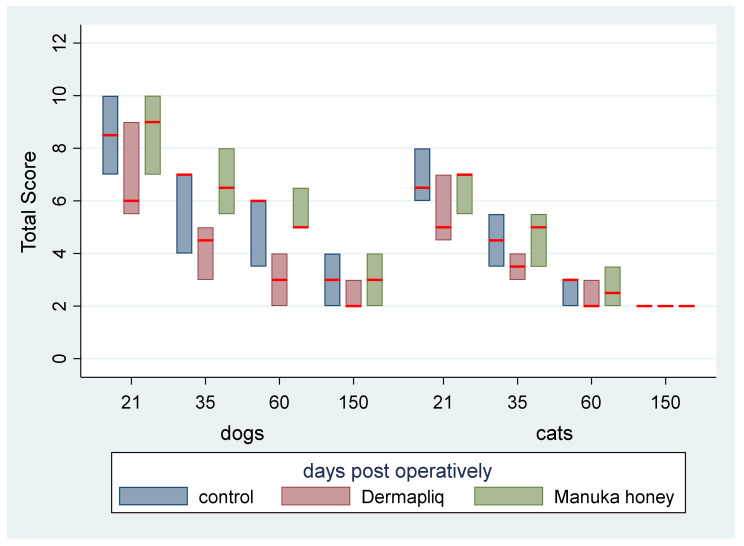
Median (red line) and interquartile range (column) of total score (without histological scores) over treatment and species and postoperative days.

The total cosmetic score, total clinical score, and USG score were added to create a total score for incision treatments’ evaluation on days 21, 35, 60, and 150, ranging from 2 to 14 and with a median value of 5. The median and interquartile range (IQR) of the total score (without histological scores) over treatment and species and postoperative days are presented in Figure 14.

According to the median regression model, adjusting for the time and species effect, a statistically significant difference between Manuka honey and Dermapliq (*p* = 0.004) was detected, suggesting that incision wounds treated with Manuka honey had a difference (higher) of 2 (95% CI: 0.65; 3.35) in their median value of the total score compared to those treated with Dermapliq. Nevertheless, there was no difference in the median value of the total score between Dermapliq and the control (*p* = 0.425) and between Manuka honey and the control (*p* = 1.00). There was a statistically significant effect of species (*p* = 0.008), suggesting that cats had a difference (lower) of −2 (95% CI: −3.46; −0.53) in their median value of the total score compared to dogs. The interaction term between species and treatment was significant, indicating that cats treated with Dermapliq treatment had a difference (higher) of 2 (95% CI: 0.48; 3.52, *p* = 0.010) in their median total score value compared to any other treatment in dogs.

### 3.5. Histological Evaluation

**Figure 15:**
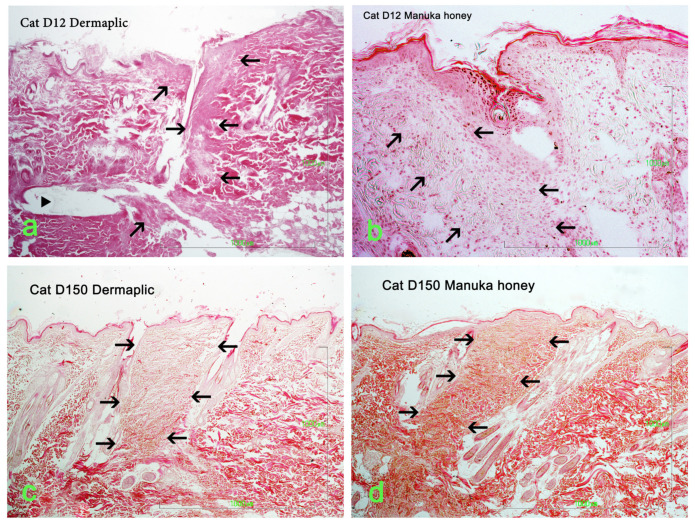
Wound area (HE stain) at the site of incision (black arrows) in a cat: (**a**) Day 12, Dermapliq (triangle: suture material); (**b**) Day 12, Manuka honey; (**c**) Day 150, Dermapliq; (**d**) Day 150, Manuka honey.

**Figure 16:**
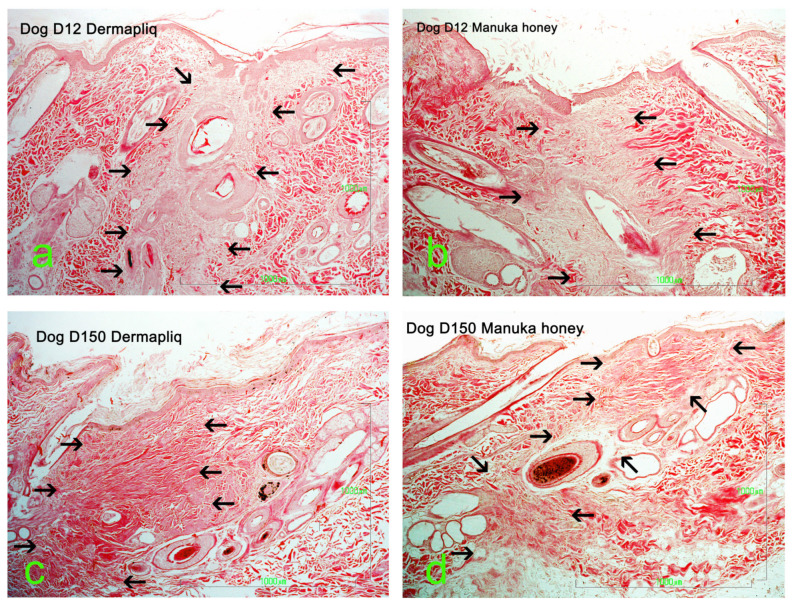
Wound area (HE stain) at the site of incision (black arrows) in a dog: (**a**) Day 12, Dermapliq; (**b**) Day 12, Manuka honey; (**c**) Day 150, Dermapliq; (**d**) Day 150, Manuka honey.

Selected images of the histological sections of the wound area are presented in Figure 15 (cat) and Figure 16 (dog).

#### 3.5.1. Edema

There was no difference in the odds of occurrence of the edema score between methods (all *p* > 0.07).

Cats were 18.3 times (95% CI: 2.66; 125) less likely to have higher versus lower edema scores compared to dogs (*p* = 0.003).

#### 3.5.2. Inflammation

Minimal or mild inflammation at the incision area was observed with both treatments and with controls. On day 12 postoperatively, infiltration by a small number of neutrophils, macrophages, and fibroblasts was observed, which is consistent with the normal healing process of the wound. The nodular accumulation of macrophages and lymphocytes at the subcutaneous tissue in a few samples was probably caused by traumatic furunculosis. Minimal to medium tissue reaction, composed of macrophages and fibroblasts, was usually observed around the suture material. On day 60 and 150 postoperatively, no inflammation was noticed. The main finding was the absence of glands and hair follicles from the wound area at the site of the incision, and the presence of thinner than normal collagen fiber bands.

A statistically significant difference between Dermapliq and the control (*p* = 0.023) was detected; there was almost a 2.3 (OR: 2.4, 95% CI: 1.13; 5.1) times less likely chance to observe higher versus lower inflammation scores with Dermapliq compared to the control. There was no difference in the odds of the inflammation score between the control and Manuka honey (*p* = 0.519) and between Manuka honey and Dermapliq (*p* = 0.099). A statistically significant effect of species was observed (*p* = 0.04); cats were almost 2.5 (OR: 2.49, 95% CI: 1.04; 6.03) more likely to have higher versus lower inflammation scores compared to dogs.

#### 3.5.3. Fibroblasts

There was no difference in the odds of higher versus lower fibroblast scores between methods (all *p* > 0.225). Cats were 2.67 (95% CI: 1.04; 6.86) times less likely to have higher versus lower fibroblast scores compared to dogs (*p* = 0.041).

#### 3.5.4. Angiogenesis

There was no difference in the odds of angiogenesis occurrence between methods (all *p* > 0.725), but a significant difference between species was detected (*p* < 0.001); cats were more likely to be associated with angiogenesis occurrence compared to dogs.

#### 3.5.5. Epidermis Thickening

**Figure 17:**
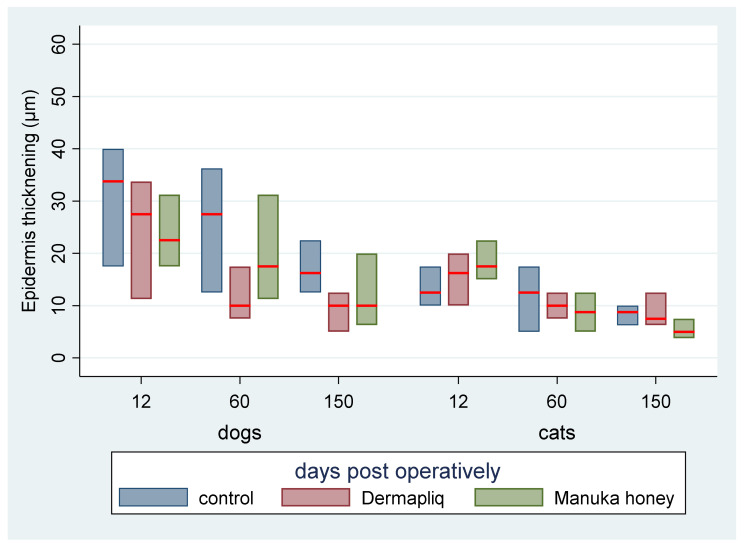
Median (red line) and interquartile range (column) of epidermis thickening by treatment and species over postoperative days.

The median and interquartile range (IQR) of epidermis thickening by treatment and species over postoperative days are presented in Figure 17.

According to the median regression models, there was not any statistically significant difference in the median value of epidermis thickening between treatments (all *p* > 0.288). Cats had a difference (lower) of −3 (95% CI: −4.8; −1.19) in their median epidermis thickening value compared to dogs (*p* = 0.001), controlling for the time and treatment effect. The epidermis thickening evaluation outcome was also transformed to a score on a scale from 0 to 3, corresponding to the 25th, the 50th, and the 75th, respectively, percentile of the distribution of total values.

#### 3.5.6. Scar Width Score

Histological scar width was transformed to a scale from 0 to 3, corresponding to the 25th, the 50th, and the 75th, respectively, percentile of the distribution of total values individually for cats’ (n_cats_ = 78, range: 15–70) and dogs’ (n_dogs_ = 104, range: 7–60) observations. For cats’ data, the 1st, 2nd, 3rd, and 4th quartiles included values of scar width ranging from 15 to 22 μm (n_Q1_ = 21), 25 to 30 (n_Q2_ = 22), 32 to 40 (n_Q3_ = 21), and 45 to 70 (n_Q4_ = 14), respectively. Meanwhile, for dogs, the 1st, 2nd, 3rd, and 4th quartiles included values of scar width ranging from 7 to 16 μm (n_Q1_ = 32), 17 to 20 (n_Q2_ = 30), 21 to 25 (n_Q3_ = 21), and 26 to 60 (n_Q4_ = 104), respectively. A statistically significant difference in the odds of scar score categories was observed between Manuka honey and the control (*p* = 0.025), adjusting for the time and species effect. Specifically, control incisions were 2.2 (95% CI: 1.10; 4.43) times more likely to have a higher versus lower scar score compared to those treated with Manuka honey. However, no difference in the odds of scar score categories between the control- and Dermapliq-treated incisions (*p* = 0.356) and between Dermapliq- and Manuka-honey-treated ones (*p* = 0.196) was detected.

Cats were 7.75 (95% CI: 2.6; 23.3) times more likely to have higher versus lower scar scores compared to dogs (*p* < 0.001).

#### 3.5.7. Total Histological Score

**Figure 18:**
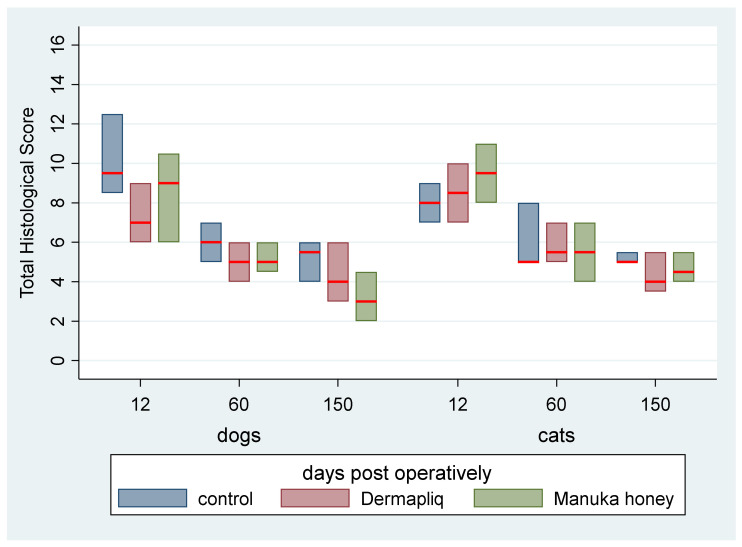
Median (red line) and interquartile range (column) of the histological total score by treatment and species over postoperative days.

Scores of each of the six histological parameters evaluated were added to create a total histological score, ranging from 2 to 14 with a median value of 6, which is presented in Figure 18.

According to the median regression model, there was not any statistically significant difference in the median value of the total histological score between Dermapliq and the control (*p* = 0.158), Manuka honey and the control (*p* = 0.156), and between Manuka honey and Dermapliq (*p* = 1.000). Moreover, no difference in the median value of the total histological score between species (*p* = 1.000) was detected. Incisions on day 60 had a difference (lower) of −3 (95% CI: −4.82; −1.08, *p* = 0.001) in their total histological score compared to those on day 12, while the respective ones on day 150 had a difference (lower) of −4 (95% CI: −5.80; −2.21, *p* < 0.001) in their total histological score compared to those on day 12. No difference was observed in the median value of the total histological score between days 60 and 150 (*p* = 0.227).

No significant treatment and day (*p* = 0.594), treatment and species (*p* = 0.579), and species and day (*p* = 1.000) interaction was observed.

## 4. Discussion

### 4.1. Dermapliq

Dermapliq is a medical device (RGTA^®^) that mimics certain properties of heparin or heparan sulphates (HSs), such as stabilizing and protecting in vitro heparin-binding growth factors and many cytokines, which play a role in cell communication [39,40]. Heparan sulphates are glycosaminoglycans that participate, by fixing on matrix proteins such as collagens, in the construction of the extracellular matrix scaffold around the cells. They are also the storage sites of growth factors, regulate their bioavailability, and ensure their action in the control of cellular homeostasis. Heparan sulphates promote cell recruitment, circulating strains via the mobilization of chemokines, and provide locally a favorable microenvironment for the implantation of these pluripotent cells [48].

However, whilst HSs are quickly destroyed during tissue damage and thus no longer ensure the protection of endogenous factors, which are themselves destroyed by the proteases released from the inflammatory phase of healing, the RGTAs replace the HS by being more resistant to degradation than the latter [48]. RGTAs therefore act mechanically, on the one hand, as structural elements of the matrix scaffolding and, on the other hand, as protectors of matrix proteins and HBGFs by protecting their degradation by proteases [48].

In animal models, RGTAs have been tested for ocular surface diseases and have shown positive effects in reducing inflammation, promoting re-epithelialization, and improving histological patterns such as edema, fibrosis, neovascularization, and inflammation [39,49]. In humans, clinical trials have demonstrated accelerated healing and reduced pain in corneal lesions resistant to other treatments [50,51,52,53].

In terms of skin wound healing, RGTAs have been shown to improve both speed and quality in preclinical models [39,54,55,56,57]. RGTAs have been used in patients with severe burn wounds, resulting in faster healing with minimal scarring, improved mobility, and pain relief [58], and on bedsores and ulcers unresponsive to any treatment, showing improvement in healing [59]. Furthermore, RGTAs have been used successfully in the treatment of ischemic skin wounds, in cases of surgical index procedures for compromised patients or ischemic wounds speeding up the healing process [58,60].

RGTAs have been found to improve the speed and quality of first-intention healing in non-contaminated wounds in humans [48,61]. In the first study by Zakine and Le Louarn [48], patients who underwent mammoplasty and centrofacial lifting received a cutaneous application of RGTA. Inflammation, pruritus, and hypertrophic scars were less frequent for the wounds treated with RGTA. Moreover, in the group that underwent centrofacial lifting, RGTA reduced discomfort, ecchymosis, scar inflammation, and edema.

In the second study by Zakine et al. [61], patients that underwent breast-reduction surgery were treated with RGTA. Closure was performed with deep dermal and superficial intradermal running sutures. Each patient had one RGTA-treated and one control breast. In the majority of cases, there was an improvement in scar symptoms on the treated side, including inflammation, pruritus, or pain. A comparison of different application regimens suggested that the best trend was obtained with a single administration of RGTA on day 0 [61].

However, the only studies on wound healing that are not based only on clinical observation were performed by Tong et al. [56], who studied the effects of RGTA on the healing of full-thickness wounds in rats. The authors found that wounds treated with RGTA had a faster closure rate, improved vasodilatory capability, and increased wound breaking strength compared to the control group and suggested that RGTA may enhance wound healing by improving microcirculation and wound strength [56]. In another study, Tong et al. [55] investigated the mechanisms of how RGTA improves cutaneous second-intention wound repair in rats and found that RGTA treatment can improve the quality of normal cutaneous wound repair and may restore impaired wound healing.

The only report on the use of RGTAs in the dog is a case series of 11 dogs treated for ulcerative keratitis associated with bullous keratopathy [62]. Martinez et al. [62] found that the corneas with recurrences of the ulcers were resolved predominantly by using RGTAs and that they can be considered a successful adjunctive therapy in the management of superficial ulcers.

#### 4.1.1. Cosmetic Evaluation

In our research, the use of RGTA Dermapliq on first-intention healing of the skin achieved a significantly better cosmetic evaluation score compared to the control in both dogs and cats, in accordance with previous reports in humans [48,61]. In dogs, the median cosmetic score with Dermapliq improved further on the 14th postoperative day and reached its best value from the 60th postoperative day onwards, whilst in cats, the best score was achieved as early as the 14th postoperative day.

#### 4.1.2. Clinical Evaluation

During clinical evaluation, overall skin thickening did not differ significantly after using Dermapliq compared to the control; however, it was less prominent until the 7th postoperative day in dogs and until the 10th postoperative day in cats while using Dermapliq. Zakine et al. [34] also found less edema in wounds closed with intradermal suturing after treatment with RGTA. Furthermore, in our study, scar width did not differ significantly between Dermapliq and the control, even if the median value was always smaller with Dermapliq in both dogs and cats. Roohi et al. [46] observed little or no scarring after using RGTA in surgical index procedures. Finally, the total clinical evaluation did not show any significant difference between Dermapliq and the control, except for the time periods days 0–27 and days 28–41, where the total clinical score was better for Dermapliq.

#### 4.1.3. Ultrasonographic Evaluation

The high-frequency ultrasonographic evaluation of wound healing has been used previously by Balomenos et al. [8,30], and proved to depict accurately the wound area and the real ultrastructure of the skin during first-intention healing, having statistically significant linear positive correlation with clinical and histological findings. Furthermore, the measurement of wound area using ultrasonography has the benefit of evaluating the extent of the wound even when a scab covers it and without the difficulty and the cost of performing a skin biopsy and histological examination.

In our study, the median wound area, as was evaluated using ultrasonographic evaluation, differed significantly when wounds were treated with Dermapliq, being smaller compared to control wounds in almost all time periods, in both dogs and cats. As wound healing proceeded, the wound area size gradually diminished, and the progressive collagen deposition altered echo intensity, making wound boundaries more complex and almost indistinguishable after the 42nd postoperative day.

#### 4.1.4. Total Score

After summing the scores of cosmetic, clinical, and ultrasonographic evaluation, the total score of each treatment was calculated, in an attempt to estimate the overall depiction of each treatment over time. The distinctive findings of each parameter with each treatment are important; however, a clinician has to choose a treatment that is overall best for first-intention healing in the dog or the cat. Therefore, a total evaluation of each treatment was attempted, even though the conclusions should be handled with some concern. In our study, total scores of the incisions treated with Dermapliq were better on all occasions, for both dogs and cats; however, these differences were non-significant.

#### 4.1.5. Histological Evaluation

From the various parameters evaluated histologically in the present study, the only statistically significant difference, between the use of Dermapliq and the controls, was noticed in the inflammation score. Wounds treated with Dermapliq showed less inflammation and it was 2.3 times more likely to have lower versus higher inflammation scores with Dermapliq, compared to the control. Tong et al. [55], in a study on second-intention wound repair in rats, also found that RGTA treatment improved inflammation resolution, but also found neovascularization, epidermal migration and proliferation, and granulation tissue formation in the wounds, which was not observed in our study, probably because we evaluated first-intention healing.

Furthermore, after combining all histological scores to generate the total histological score, in an attempt to estimate the overall depiction of each treatment on histological findings, no statistically significant differences were found between Dermapliq-treated and control wounds.

### 4.2. Manuka Honey

The medicinal importance of honey has been documented in the world’s oldest medical literature, and since ancient times, it has been known to possess antimicrobial properties as well as wound-healing activity [37]. There are many reports of honey being very effective in dressing wounds, burns, skin ulcers, and inflammations [63].

The healing property of honey is attributed to the fact that it offers antibacterial activity and keeps wounds moistened, and its high viscosity helps to provide a protective barrier to prevent infection; thus, the antibacterial properties of honey speed up the growth of new tissue to heal the wound [64]. Its mechanism of action may also be related to its low pH level and its high sugar content (high osmolarity), which is enough to hinder the growth of microbes. Its immunomodulatory property is also relevant to wound repair. The antimicrobial activity in most types of honey is due to the enzymatic production of hydrogen peroxide (H_2_O_2_) [37].

The unpredictable antibacterial activity of non-standardized honey may, however, hamper its introduction as an antimicrobial agent due to variations in the in vitro antibacterial activity of various honeys. At present, several honeys are sold with standardized levels of antibacterial activity, of which the best known is Manuka honey [37]. Manuka honey is the monofloral product of *Leptospermum scoparium*, a New Zealand native plant, said to possess “non-peroxide anti-bacterial activity” [65], meaning it displays significant antibacterial effects even when the hydrogen peroxide activity is blocked.

The beneficial role of Manuka honey is attributed to its antibacterial property with regards to content of non-peroxide components, i.e., the presence of phytochemical components [65,66] such as glyoxal (GO), 3-deoxyglucosulose (3-DG), and methylglyoxal (MGO), which have been extensively studied in Manuka honey [65,67,68]. Kilty et al. [36] found that methylglyoxal is an effective antimicrobial agent against *Pseudomonas aeruginosa* and *Staphylococcus aureus* biofilms in vitro. Biofilms may persist in wounds and impair healing [29]. In fact, Manuka honey at 50% was shown to cause ‘‘significant partial detachment’’ of *Proteus* biofilms after 24 h [29].

Manuka honey is also capable of stimulating monocytes, the precursors of macrophages, to secrete TNF-α [69,70]. On the other hand, glycosylated proteins can induce TNF-α secretion by macrophages, and this cytokine is known to induce the mechanism of wound repair. Furthermore, the ability of honey to reduce “reactive intermediates release” [69] may well limit tissue damage by activated macrophages during wound healing.

Finally, propolis, another component of honey, contains chiefly flavonoids, phenolic acids, and their esters, which may also contribute to its immuno-stimulant properties [34].

In the literature, there are various studies on the use of Manuka honey on surgical and traumatic wounds, most of them in humans [29]. Manuka dressings have been reported to cause a significant decrease in wounds’ size, healing time, and pain levels when used for the treatment of various wounds [71,72,73]. However, there are studies questioning the effectiveness of Manuka honey on wound management [29,74].

Furthermore, several other clinical trials and experimental control studies investigated various types of honey as agents for wound healing enhancement using laboratory animals such as rats, mice, and rabbits. Overall, a careful review of the results of these studies reveals promising beneficial effects of honey or honey products on wound healing [75].

Various types of honey have also been investigated in dogs [76,77] and they seem to accelerate healing and surgical wound closure and decrease the level of infection. In cats, L-Mesitran^®^ Soft medical honey wound gel has been used on contaminated wounds that healed by second intention [78,79]. The authors concluded that the treatment of wounds with medical honey had a positive impact on wound healing, and the cosmetic appearance was minimally altered due to a minimal amount of scar tissue and hair regrowth. However, in either of these studies, other types of honey, instead of Manuka, were used.

The only studies that featured histological evaluation after the use of Manuka honey on the wound area are those of Repellin et al. [38] in dogs, Singh et al. [80] in mice, and Bischofberger et al. [81] in horses. All these studies evaluate the effect of Manuka honey on acute, full-thickness wound healing by second intention and not their effect on sutured surgical wounds healing by first intention.

Repellin et al. [38] evaluated the effect of a proprietary Manuka honey essential oil hydrogel on acute, full-thickness wounds healed by second intention in dogs and reported that its application may be beneficial in the early proliferative stage of wound healing. However, in their research, Manuka honey was not used by itself, so their findings cannot be attributed only to Manuka honey [38].

Singh et al. [80] developed scar preventive dressings containing Manuka honey for wound care and used them in a mice model. They reported that wound healing using Manuka honey was achieved through the suppression of inflammatory cells and the proliferation of epithelial and fibroblast cells. They also observed scar formation with a thick epidermis due to uncontrolled fibroblast proliferation, with numerous collagen tissues along with angiogenesis and with the presence of a few inflammatory cells. They concluded that dressings containing Manuka honey are capable of delivering fast, smooth, and scar-free wound healing; however, due to species differences and the type of the wound and healing method (second intention), their results might not be applied to the first-intention healing in the dog and the cat [80].

Bischofberger et al. [81] investigated the effect of Manuka honey gel on the transforming growth factor β1 and β3 concentrations, bacterial counts, and histomorphology of contaminated full-thickness skin wounds healed by second intention in equine limbs. They found that the daily application of 66% Manuka honey gel to wounds had no significant effect on TGF-β1 and TGF-β3 concentrations. Furthermore, it had no apparent effect on bacterial counts during the first 10 days after wound creation, which was surprising, given the well-established antibacterial activity of Manuka honey. However, they also found that Manuka honey gel decreased wound inflammation; increased angiogenesis, fibrosis, collagen organization, and epithelial hyperplasia; and improved epithelialization [81].

Another study by Bischofberger et al. [82], albeit without histological evaluation, evaluated the effect of short- and long-term treatment on contaminated and non-contaminated open wounds in horses, which were left to heal by second intention. Their results indicated that all wounds treated with Manuka honey healed faster, compared to control wounds. Both these studies evaluated the application of Manuka honey in another species, under very different conditions (open, contaminated wounds), so their findings might not be applicable in dogs and cats [82].

Tsang et al. [83] compared the effect of the application of Manuka honey with a generic multifloral honey on equine wound healing variables and found that, in clean uncontaminated wound healing by second intention, UMF20 was superior to both commercial food-grade multifloral honey and UMF5 as a topical treatment. However, in their model, the beneficial effects were limited to decreasing the total healing time, and not to other wound healing variables, and the difference was modest and likely to be clinically insignificant [83].

#### 4.2.1. Cosmetic Evaluation

In our study, the use of Manuka honey on first-intention healing of the skin did not show a significantly better cosmetic evaluation score compared to the control (*p* = 0.052), although it achieved better outcomes in most evaluations. Heidari et al. [84], who examined the use of a different type of honey on post-Cesarean-section wounds, found no statistically significant difference in scar formation when compared to controls. In other studies, where Manuka honey or other types of honey were used, the treatment was used in second-intention healing, in compromised wounds or in non-healing wounds, where the cosmetic outcome was not a priority and no relative information was given.

In dogs, the median cosmetic score with Manuka honey improved on day 21 and then stayed stable, whilst in cats, the score improved on day 14, achieving a better value than dogs afterwards.

#### 4.2.2. Clinical Evaluation

During clinical evaluation, overall skin thickening differed significantly after using Manuka honey, being 0.30 mm higher in its median value compared to the control.

More specifically, it was significantly more prominent on days 11–19 and 20–24 postoperatively, both in dogs and cats, whilst no statistically significant difference was found in the other time periods. On the contrary, Nikpour et al. [85], when using 25% honey gel (containing coriander and Goat’s thorn honey) on post-Cesarean-section sutured wounds in women, found significantly lower wound edema on days 7 and 14 of treatment, but this may be due to the use of a different type of honey in a different species.

Clinically estimated scar width did not differ significantly between Manuka honey and the control overall; the median value was always higher than the control in dogs but not in cats. However, on days 20–34 postoperatively, the differences between Manuka honey and the control were significant, and incision wounds treated with Manuka honey had a difference of 0.4 in the median value of scar width compared to the control, probably because of the more prominent skin thickening at this time period with Manuka honey treatment.

As a consequence, the total clinical evaluation showed a significantly worse score with Manuka treatment compared to the control; incision wounds treated with Manuka honey had a difference in the median total clinical score of 0.94 compared to the control. This difference was more prominent, and statistically significant, on days 1–19 and 42–150.

#### 4.2.3. Ultrasonographic Evaluation

In our study, the median wound area, as evaluated using ultrasonography, differed significantly (being smaller) when wounds were treated with Manuka honey, with the difference being more apparent in dogs. This result seems to contradict clinical findings, where Manuka honey treatment led to a worse clinical score. However, ultrasonography evaluates the real wound area and is not influenced by subcutaneous edema, something that happens when skin thickening is measured clinically.

As wound healing proceeded, the wound area size gradually diminished, and the progressive collagen deposition altered echo intensity, making wound boundaries more complex and almost indistinguishable after day 42.

#### 4.2.4. Total Score

In our study, the median total scores of the incisions treated with Manuka honey were not different from those of controls, and no statistically significant differences were found. In dogs, Repellin et al. [38], who evaluated the effect of a proprietary Manuka honey essential oil hydrogel (HoneyCure^®^) on acute wounds healed by second intention, found that the only difference in favor of HoneyCure was the percent epithelialization of wounds, something that could not be depicted in our research since it was on first-intention healing. Repellin et al. [38] concluded that their study did not provide evidence to support the application of HoneyCure in small, acute wounds in healthy dogs, as was the case in our study, a conclusion that is in agreement with our results.

#### 4.2.5. Histological Evaluation

In our research, the histological findings on edema, inflammation, the presence of fibroblasts, and angiogenesis did not differ between Manuka-honey-treated wounds and controls. As far as inflammation is concerned, the lack of difference can be explained by the fact that our treatment trial was performed on small, clean, acute wounds in healthy dogs and cats, closed by first intention, and the wounds were bandaged and the animals received prophylactic antimicrobial therapy, so no inflammation was observed in control wounds either. Repellin et al. [38], who evaluated the effect of a proprietary Manuka honey essential oil hydrogel on acute wound healing by second intention in dogs, did not find any difference in the histological scores between the treatment group and controls. Singh et al. [80] examined histological wounds in mice healed by second intention, treated with bionanocomposite dressings containing Manuka honey, on the 21st post-wounding day. They reported that “wound healing using Manuka honey was achieved by suppression of inflammatory cells and the proliferation of epithelial and fibroblasts cells”, however, without any other information. Bischofberger et al. [81], who investigated the effect of Manuka honey gel on second-intention wound healing on contaminated wounds in horses, found that it decreased wound inflammation (on days 7 and 10), increased angiogenesis as early as 2 days after wounding (on days 2, 7, and 10), increased fibrosis and collagen organization (on day 7), and increased epithelial hyperplasia (on days 7 and 10), thus improving epithelialization. Their findings, however, cannot be compared to this study, due to the contamination of the wounds and the different species used.

In our study, epidermis thickening in wounds treated with Manuka honey did not differ significantly from those left untreated and was on most occasions lower than controls. This contradicts the findings of the mice model of Singh et al. [80], who observed scar formation with a thick epidermis due to uncontrolled fibroblast proliferation with numerous collagen tissues along with angiogenesis and with the presence of few inflammatory cells. This is probably because of the different species and the second-intention healing in their research.

In our study, the only histological parameter that was found to differ significantly between Manuka-treated and untreated wounds was the scar width score. Specifically, control incisions were 2.2 times more likely to have higher versus lower scar scores compared to those treated with Manuka honey.

Finally, after combining all histological scores to generate the total histological score, in an attempt to estimate the overall depiction of each treatment on histological findings, no statistically significant differences were found between Manuka-honey-treated and control wounds.

### 4.3. Comparison between Dermapliq and Manuka Honey

Another objective of the present study was to determine which treatment, Dermapliq or Manuka honey, has a more positive effect on first-intention healing in dogs and cats. For the comparison between the treatments, all the cosmetic, clinical, ultrasonographic, and histological data on the cutaneous wound healing that were obtained from the cats and dogs during this study were used. The median value of each measurement was the dependent variable in the quantile regression model, while treatment was the respective independent one.

The cosmetic evaluation revealed a statistically significant difference between Dermapliq and Manuka honey, with Dermapliq achieving better cosmetic scores.

Clinical evaluation also revealed statistically significant differences in skin thickening, with Dermapliq achieving less intense thickening on days 0 to 34 compared to Manuka honey, and in scar width, with Dermapliq achieving smaller scar width, especially on days 20 to 34. As a result, the total clinical score differed significantly between Dermapliq and Manuka honey, with Dermapliq being better.

On the contrary, ultrasonographic evaluation and histological evaluation did not reveal any significant differences between the two treatments.

However, when the total cosmetic score, total clinical score, and USG score were added to create a total score for treatments’ evaluation on days 21, 35, 60, and 150, a statistically significant difference between Manuka honey and Dermapliq was detected, with Dermapliq achieving a better score.

In the present study, in which first-intention healing was evaluated, Dermapliq proved to be a better choice in achieving favorable wound healing compared to Manuka honey in dogs and cats. This may be explained by the fact that the two treatments affect wound healing in different ways. Dermapliq, which is a device that helps healing by stabilizing and protecting heparin-binding growth factors and matrix proteins, seems to be more effective on first-intention healing in clean surgically induced wounds in healthy animals than Manuka honey, which is usually used for its antimicrobial action in infected, compromised non-healing wounds.

### 4.4. Comparison between Dogs and Cats

Studies have shown that there are variations in wound healing between dogs and cats [2,86]. The anatomy of the feline skin is similar to the canine; however, the feline skin is looser, more pliable, and more mobile over most of the body surface than the canine skin, and the epidermis, dermis, and subcutis are slightly thinner in cats than in dogs [87,88]. Cats have reduced cutaneous perfusion, less granulation tissue production and epithelialization, weaker tissue contraction, and weaker healed tissue strength compared to dogs. Additionally, granulation tissue formation in cats follows a different pattern compared to in dogs [9,11].

Dogs are known to have a more robust early inflammatory response compared to cats, which may contribute to these differences [9].

The variations in wound healing between dogs and cats are believed to be influenced by differences in their cutaneous angiosomes, i.e., specific tissue areas with distinct vascular supply (vascular territories). Taylor and Minabe [89], who extensively studied angiosomes of mammals, concluded that dogs have a greater number of well-distributed cutaneous perforating vessels. Cats, on the other hand, have a smaller number of cutaneous perforators that are more widely distributed in two major lines along the trunk [11,89]. They also have a relatively low density of tertiary and higher-order vessels, particularly in the trunk.

In dogs, granulation tissue formation starts at the base of the wound and can spread throughout the entire wound surface, giving it a deep red appearance. On the other hand, in cats, granulation tissue forms at the edge of the wound and moves toward the center with a paler hue [9,10,11].

A previous study [9] reported that incisions in cats, on the 7th post-traumatic day, had only half the breaking strength compared to those in dogs. This was attributed to a delay in collagen production and to the presence of immature collagen in the wound bed. In cats, the phenomenon of pseudohealing is observed, where the incision may appear to have healed normally upon visual inspection, but normal physiological stresses can result in dehiscence and reveal inadequate healing of the underlying tissues. Thus, it may be advantageous to keep sutures in place for a longer duration than the usual 10–14 days after wound closure [9].

There are only two published studies describing cutaneous wound healing in cats in comparison to dogs [9,11]. They evaluated first-intention wound healing by measuring the breaking strength of sutured linear cutaneous wounds and by evaluating cutaneous vascular perfusion with Laser Doppler flowmetry. They found that wound breaking strength in cats is significantly inferior compared to dogs and hypothesized that this difference in early first-intention healing reflects significantly lower collagen production in cats compared to dogs.

So, another objective of this study was to determine if the treatment with Dermapliq and Manuka honey influences first-intention healing differently in dogs compared to cats, and if this difference is time-dependent. For the comparison between cats and dogs, all the cosmetic, clinical, ultrasonographic, and histological data on the cutaneous wound healing obtained during this study were used.

#### 4.4.1. Cosmetic Evaluation

In our study, a statistically significant difference in the odds of the total cosmetic score was observed between species, as cats were 11.6 times less likely to have higher versus lower cosmetic sum scores compared to dogs, meaning that cats had a better cosmetic score.

#### 4.4.2. Clinical Evaluation

During clinical evaluation, overall skin thickening differed significantly between dogs and cats, with cats achieving a lower median value of skin thickening compared to dogs, something that is expectable since cats’ epidermis, dermis, and subcutis are slightly thinner than those of dogs [87]. When skin thickening was tested along with healing, the difference between cats and dogs was significant on days 0–10 and 11–19, being −0.51 and −0.3, respectively, whilst afterwards, the difference was not significant.

Cats also had a statistically significant difference (lower) in the overall median value of scar width compared to dogs. When scar width was tested along with healing, the difference between cats and dogs was significant in all periods; the difference started at −0.8 for the first 20 days and declined gradually to −0.47 at the end of the experiment. Furthermore, on days 20–34, a statistically significant interaction was detected between species and treatment, meaning that the effect of treatment on scar width was different in cats compared to dogs. The effect of Manuka honey treatment was higher (greater) in cats than in dogs by an additional difference of −0. 4 in the median value of scar width, compared to the control, and by an additional difference of −0.5, compared to Dermapliq, meaning that Manuka honey achieved better results as far as scar width is concerned in cats.

The total clinical evaluation overall did not show any significant difference between species. However, on days 1–19, the species effect was significant and cats had a difference of −1.7 in their total clinical score compared to dogs. On days 42–150, there was also a statistically significant difference between species; cats had a difference of −1.58 in their total clinical score compared to dogs.

#### 4.4.3. Ultrasonographic Evaluation

In our study, the median wound area, as was evaluated using ultrasonography, did not differ significantly overall. However, on days 10–17, a statistically significant difference between species was found; cats had a difference of −1.03. The difference was also significant afterwards, being −1.43 (days 21–35) and −0.16 (days 42–150).

The interaction between species and treatment was significant after day 42, suggesting that non-treated wounds in cats had a lower median value of 0.35 units.

#### 4.4.4. Total Score

After summing the scores of cosmetic, clinical, and ultrasonographic evaluation, the total score of each treatment was calculated. A statistically significant effect of species was found, with cats achieving a significant difference (lower) of −2 units in their median value of the total score compared to dogs. The interaction term between species and treatment was significant, indicating that cats treated with Dermapliq had a difference (higher) of 2 units in their median total score value compared to any other treatment in dogs.

#### 4.4.5. Histological Evaluation

All parameters evaluated histologically in the present study differed between cats and dogs.

Cats showed significantly less edema when compared to dogs and were 18.3 times less likely to have higher versus lower edema scores compared to dogs.

A statistically significant effect of species was also observed in the inflammation score, where cats were almost 2.5 times more likely to have higher versus lower inflammation scores compared to dogs. Our findings are in contrast with the suggestion of Bohling et al. [9] that cats might have a less robust early inflammatory response compared to dogs.

Furthermore, our study found that cats were 2.67 times more likely to have lower versus higher fibroblast scores compared to dogs, the difference being significant. This finding is in accordance with Bohling et al.’s hypothesis that the low wound breaking strength in early first-intention healing reflects significantly lower collagen production in wounds in cats compared to dogs [11]. However, we found that cats were more likely to have higher versus lower angiogenesis scores compared to dogs, whilst Bohling et al. [11] did not find any significant temporal differences in LDPI values or percent change between cats and dogs on or after day 7 postoperatively. Also, in the present study, cats had a difference (lower) of −3 in their median epidermis thickening value compared to dogs.

Finally, no difference in the median value of the total histological score between species was detected.

## 5. Conclusions

The use of Dermapliq in first-intention wound healing achieved a significantly better cosmetic evaluation score and better total clinical score (from the 20th to the 41st postoperative day), compared to the control, in both dogs and cats. The ultrasonographically estimated wound area was also significantly smaller compared to the control when wounds were treated with Dermapliq. Wounds treated with Dermapliq showed histologically less inflammation compared to the control.

The use of Manuka honey on first-intention healing of the skin did not show a significantly better cosmetic score compared to the control. Skin thickening was significantly higher after using Manuka honey compared to the control and so was the total clinical score. However, the median wound area, as was evaluated ultrasonographically, was significantly smaller when wounds were treated with Manuka honey, the difference being more apparent in dogs.

Dermapliq was proven to be a better choice in achieving favorable wound healing compared to Manuka honey in dogs and cats, showing statistically significant differences in cosmetic evaluation, skin thickening, scar width, total clinical score, and total score.

In the present study, cats had a statistically better cosmetic score and significantly lower skin thickening and scar width compared to dogs. A statistically significant effect of species was found, with cats achieving a significantly lower total score compared to dogs. Also, cats treated with Dermapliq had a higher total score value compared to any other treatment in dogs. Histologically, cats showed significantly less edema, a higher inflammation and angiogenesis score, and a lower fibroblast and epidermis thickening score when compared to dogs.

## Data Availability

The data presented in this study are available in the article.

## Supplementary Materials

The following supporting information can be downloaded at: https://www.mdpi.com/article/10.3390/vetsci11020064/s1, Figure S1: Wounds’ photographs from cat 6; Table S1: Scoring system of clinical evaluation; Table S2: Scoring system of histological evaluation.
