# Supplementary material for: First-Intention Incisional Wound Healing in Dogs and Cats: A Controlled Trial of Dermapliq and Manuka Honey
Source: Vet Sci. 2024 Feb 1;11(2):64. doi: 10.3390/vetsci11020064 (PMC10892332; doi:10.3390/vetsci11020064)
Supplement: Supplementary file 1 [file vetsci-11-00064-s001.zip › vetsci-2776673-supplementary.pdf]

Supplementary Materials

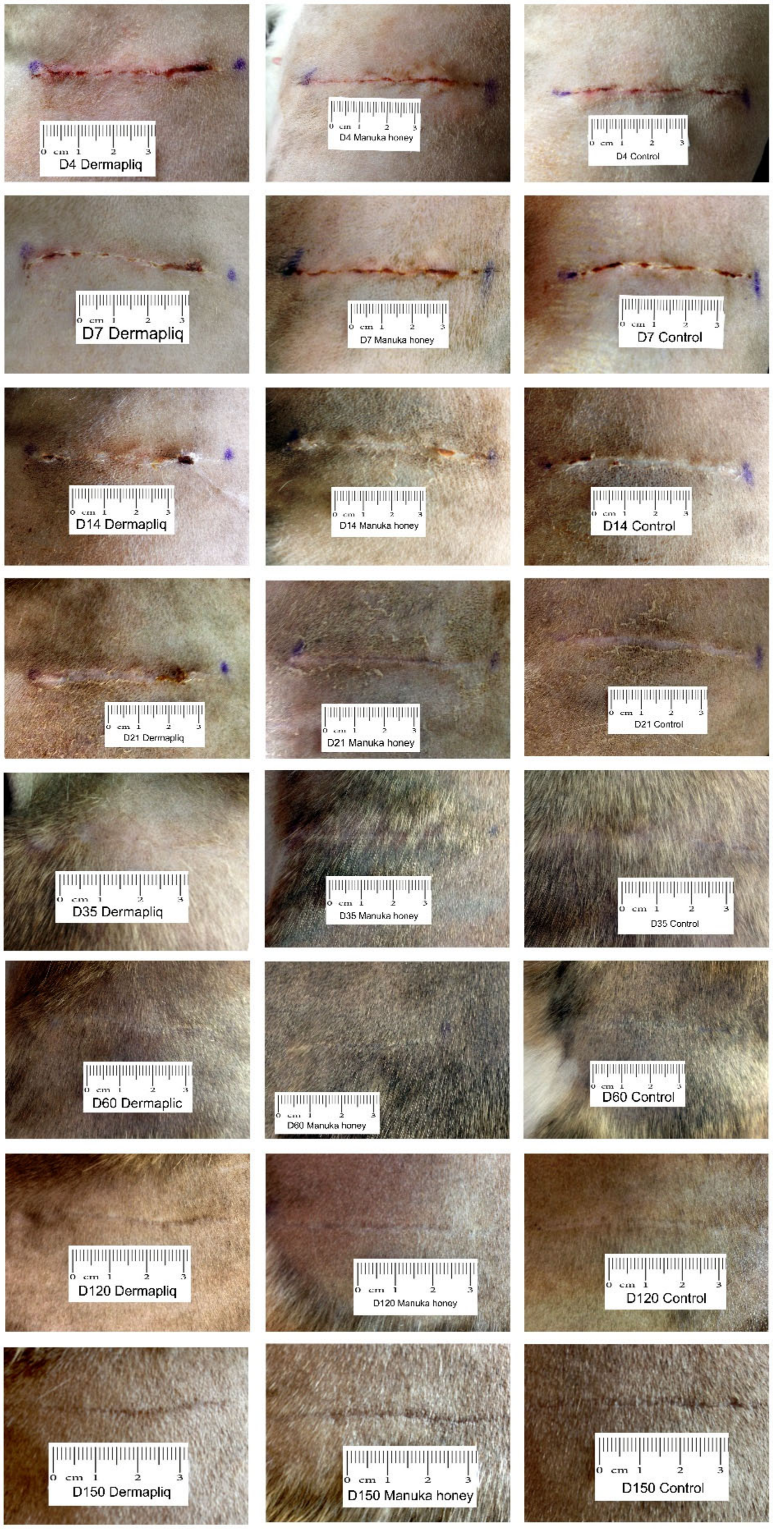

Figure S1. Photographs of the wounds in cat 6.

Table S1. Scoring system of clinical evaluation.

CLINICAL EVALUATION	Score 0	Score 1	Score 2	Score 3
Skin thickening	measured with a tuberculin skin testing ruler at the central part of the incision, in mm			
Scar width	measured with an electronic caliper, in the central part of the incision, in mm			
Abscessation or inflammation	absence	mild inflammation	1-2 microabscesses	intense inflammation, or more than 3 microabscesses
Exudate	absence	serosanguineous	seropurulent	purulent
Wound dehiscence			Length in cm	

Table S2. Scoring system of histological evaluation.

HISTOLOGICAL EVALUATION	Score 0	Score 1	Score 2	Score 3
Oedema [32]	absence of oedema, normal,	slight separation of cells and collagen from each other in the wound tissue by non-stained or poorly stained acellular material	separation of approximately 30 to 50 μm by this acellular material,	separation of > 50 μm)
Inflammation [45]	<3 cells/field	3-10 cells/field	11-30 cells/field	>31 cells/field
	evaluated by scoring neutrophils, eosinophils, macrophages, lymphocytes, plasma cells, and mast cells detected in 10 high power fields (HPF) (400x)			
Epithelial thickness [45]	the difference between adjacent healthy epidermis thickness and epidermis thickness at the scar			
Scar width [7]	in μm, then transformed to a score in a scale from 0 to 3, corresponding to the 25th, the 50th, and the 75th, respectively, percentile of the distribution of total values			
Presence of fibroblasts [32,44]	<3 fibroblasts/field 400x	3-10 fibroblasts/field 400x	11-30 fibroblasts/field 400x	>31 fibroblasts/field 400x
Angiogenesis [32,44]	<3 capillary buds/field 400x	3-10 capillary buds/field 400x	11-30 capillary buds/field 400x	>31 capillary buds/field 400x

References

7 Gouletsou, P.G.; Prassinos, N.N.; Papazoglou, L.G.; Kostoulas, P.; Galatos, A.D. Comparison of continuous intradermal with simple interrupted suture pattern: an experimental study in dogs. *Top Companion Anim. Med.* **2020**, *41*, 100454, doi:10.1016/j.tcam.2020.100454.

32 Gillette, R.L.; Swaim, S.F.; Sartin, E.A.; Bradley, D.M.; Coolman, S.L. Effects of a bioactive glass on healing of closed skin wounds in dogs. *Am. J. Vet. Res.* **2001**, *62*, 1149–1153, doi:10.2460/ajvr.2001.62.1149.

44 Winkler, J.T.; Swaim, S.F.; Sartin, E.A.; Henderson, R.A.; Welch, J.A. The effect of a porcine-derived small intestinal submucosa product on wounds with exposed bone in dogs. *Vet. Surg.* **2002**, *31*, 541–551, doi:10.1053/jvet.2002.34669.

45 Kirpensteijn, J.; Maarschalkerweerd, R.J.; Koeman, J.P.; Kooistra, H.S.; van Sluijs, F.J. Comparison of two suture materials for intradermal skin closure in dogs. *Vet. Q* **1997**, *19*, 20–22, doi:10.1080/01652176.1997.9694732.
